# Dual Role of Topoisomerase II in Centromere Resolution and Aurora B Activity

**DOI:** 10.1371/journal.pbio.0060207

**Published:** 2008-08-26

**Authors:** Paula A Coelho, Joana Queiroz-Machado, Alexandre M Carmo, Sara Moutinho-Pereira, Helder Maiato, Claudio E Sunkel

**Affiliations:** 1 Instituto de Biologia Molecular e Celular (IBMC), Porto, Portugal; 2 Faculdade de Ciências da Saúde, Universidade Fernando Pessoa, Porto, Portugal; 3 Laboratory of Cell and Molecular Biology, Faculdade de Medicina, Universidade do Porto, Porto, Portugal; 4 Instituto de Ciências Biomédicas de Abel Salazar (ICBAS), Universidade do Porto, Porto, Portugal; Dana Farber Cancer Institute, United States of America

## Abstract

Chromosome segregation requires sister chromatid resolution. Condensins are essential for this process since they organize an axial structure where topoisomerase II can work. How sister chromatid separation is coordinated with chromosome condensation and decatenation activity remains unknown. We combined four-dimensional (4D) microscopy, RNA interference (RNAi), and biochemical analyses to show that topoisomerase II plays an essential role in this process. Either depletion of topoisomerase II or exposure to specific anti-topoisomerase II inhibitors causes centromere nondisjunction, associated with syntelic chromosome attachments. However, cells degrade cohesins and timely exit mitosis after satisfying the spindle assembly checkpoint. Moreover, in topoisomerase II–depleted cells, Aurora B and INCENP fail to transfer to the central spindle in late mitosis and remain tightly associated with centromeres of nondisjoined sister chromatids. Also, in topoisomerase II–depleted cells, Aurora B shows significantly reduced kinase activity both in S2 and HeLa cells. Codepletion of BubR1 in S2 cells restores Aurora B kinase activity, and consequently, most syntelic attachments are released. Taken together, our results support that topoisomerase II ensures proper sister chromatid separation through a direct role in centromere resolution and prevents incorrect microtubule–kinetochore attachments by allowing proper activation of Aurora B kinase.

## Author Summary

Successful cell division requires that chromosomes are properly condensed and that each sister chromatid is self-contained by the time the sister pairs are segregated into separate daughter cells. It is also essential that the kinetochores at the centromeres of each pair of sister chromatids bind microtubules from opposite spindle poles. Topoisomerase II is a highly conserved enzyme that removes interlinks from DNA and is known to be essential to proper chromosome segregation during cell division. In this work, we have used state-of-the-art four-dimensional fluorescent microscopy to follow progression through mitosis in living cells depleted of topoisomerase II. We find that when the enzyme is absent, the two sister centromeres do not separate, and chromosomes missegregate. Moreover, the inappropriate centromere structure that results prevents the correct activation of the Aurora B kinase, which forms part of a regulatory mechanism that monitors correct segregation of chromosomes; as a result, cells exit mitosis abnormally.

## Abstract 3

Analysis of cells lacking topoisomerase II reveals that the enzyme has an essential role in the segregation of chromosomes, and specifically centromeres, at anaphase-telophase of mitosis: it prevents non-disjunction and allows activation of the Aurora B kinase, so as to correct improper attachments between microtubules and the kinetochore.

## Introduction

Ordered segregation of the genome during cell division requires bipolar attachment to spindle microtubules [1] and maintenance of sister chromatid cohesion until anaphase onset [2]. Cohesin provides a physical link between sister chromatids, and cleavage of cohesin subunits results from separase activation after the spindle assembly checkpoint (SAC) is satisfied [3]. However, before segregation occurs, proper chromosome condensation and sister chromatid resolution must be completed. The condensin complex has been shown to play a key role in these processes by organizing an axial structure where topoisomerase II (TOPO II) localizes and decatenates entangled DNA strands that result from replication or transcription [4,5]. Indeed, the requirement of TOPO II activity in mitosis has been amply documented. In Saccharomyces cerevisiae, circular DNA molecules accumulate as catenated dimers in top2 mutants [6], and TOPO II activity prevents nondisjunction and DNA breakage during mitosis [7–9]. Injection of antibodies against TOPO II in *Drosophila* embryos [10], the addition of TOPO II inhibitors or RNA interference (RNAi) in mammalian culture cells and *Xenopus* extracts [11–14] caused severe defects in chromosome segregation during anaphase. More specifically, TOPO II activity has been suggested to affect normal centromere structure [15] where the protein normally accumulates in its catalytically active form [15–20]. These data strongly suggest that prior to segregation, TOPO II has a general role in promoting the resolution of sister chromatids. However, how this correlates with TOPO II activity at the centromeres remains a critically unanswered question.

## Results

### Characterization of Cells Depleted of TOPO II or Inhibited with Anti–TOPO II Drugs

**Figure 1:**
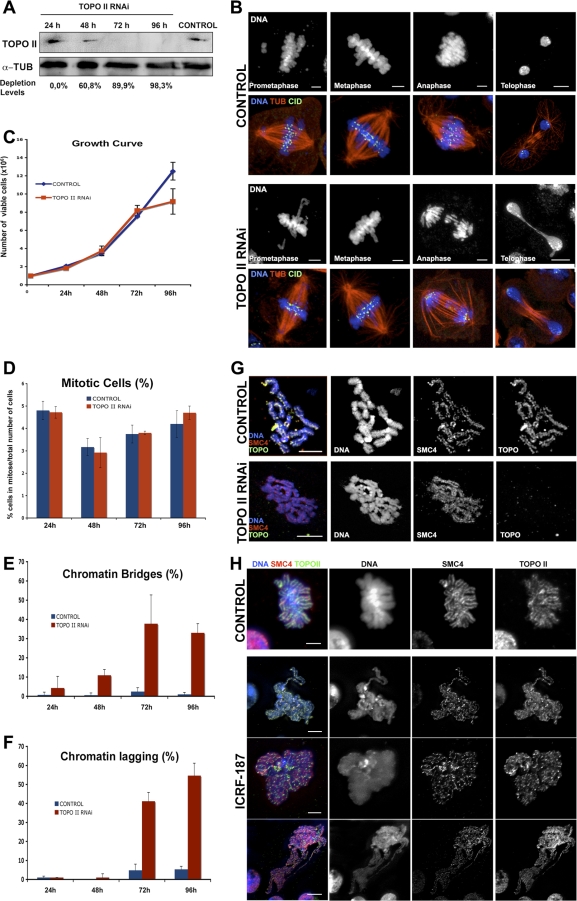
Analysis of S2 *Drosophila* Cells Depleted of TOPO II by dsRNAi (A) Depletion of TOPO II over time was monitored by western blot. α-Tubulin (α-TUB) was used as a loading control. TOPO II protein was barely detectable 72 h after the addition of dsRNA. (B) Representative images from immunofluorescence of the control and TOPO II–depleted mitotic cells stained for CID (green), α-tubulin (TUB; red), and DNA (blue). TOPO II–depleted cells progress through mitosis, exhibiting lagging chromatids or telophase bridges (DNA individual channel above). (C) Proliferation curve of control cells and of TOPO II dsRNAi-treated S2 cells throughout the experiment. Cell proliferation was clearly affected 72 h after the addition of dsRNA. (D) Mitotic index quantification shows no significant differences between control and TOPO II–depleted cells through the time course of the experiment. (E and F) The phenotype associated with TOPO II depletion, both chromatin bridges (E) and chromatin lagging (F), start to become consistently observed 72 h after the addition of the dsRNAi. This time point correlates to higher levels of depletion quantified by western blotting and with the beginning of an effect in cell cycle proliferation. (G) Immunolocalization of SMC4 (red), TOPO II (green), and DNA (blue) on S2 mitotic cells treated with hypotonic shock were performed in control (Top) or TOPO II–depleted (bottom) cells. SMC4 localizes to the central axis of the TOPO II–depleted chromosomes similar to control cells, suggesting that condensin localization to the central axis of chromosomes is independent of TOPO II. (H) Immunolocalization of SMC4 (red) and TOPO II (green) in S2 *Drosophila* cells control and treated with 50 μg/ml ICRF-187. Compared to the control, the chromosomes of cells treated with the TOPO II inhibitor are longer extended. During anaphase, extensive bridges are also observed. Although the chromosome morphology is altered after incubation with the inhibitor, both SMC4 and TOPO II are still associated with chromatin. Scale bar represents 5 μm.

To study the function of TOPO II during mitosis, we first analyzed the consequences of depleting the enzyme by RNAi or treating *Drosophila* S2 cells with specific inhibitors (Figure 1). Significant levels of TOPO II depletion were obtained by RNAi treatment as shown by western blot analysis in which the protein is barely detectable after 72 h (Figure 1A). However, we found that these cells apparently progress normally through early stages of mitosis but show severe segregation defects during anaphase and telophase, and cell proliferation is significantly inhibited without altering the mitotic index (Figure 1B–1D). Quantification of chromosome segregation abnormalities shows that after long RNAi treatment, a significant proportion of cells display either chromatin bridges or lagging chromatids during anaphase (Figure 1B, 1E, and 1F). Immunofluorescence analysis of chromosome morphology with antibodies against condensin subunits reveals that depletion of TOPO II does not significantly affect mitotic chromosome structure (Figure 1G). Cells were also treated with the TOPO II inhibitor ICRF-187, a bisdioxopiperazine-type chemical that has been shown to interfere with the catalytic activity of TOPO II [21]. However, treatment of cells with ICRF-187 results in a more pronounced alteration in chromosome structure (Figure 1H). The exact role of TOPO II in mitotic chromosome structure remains highly debatable. This is due to the fact that the use of different procedures to disrupt TOPO II function and localization in several model organisms has led to conflicting results [22]. Moreover, previous studies have shown that TOPO II inhibitors may also result in the activation of the G2 checkpoint because they block the activity of the enzyme in different conformation states [23]. Therefore, we have resorted to depleting TOPO II by RNAi for most of our study.

### TOPO II Has an Essential Role in Centromere Disjunction during Mitosis

**Figure 2:**
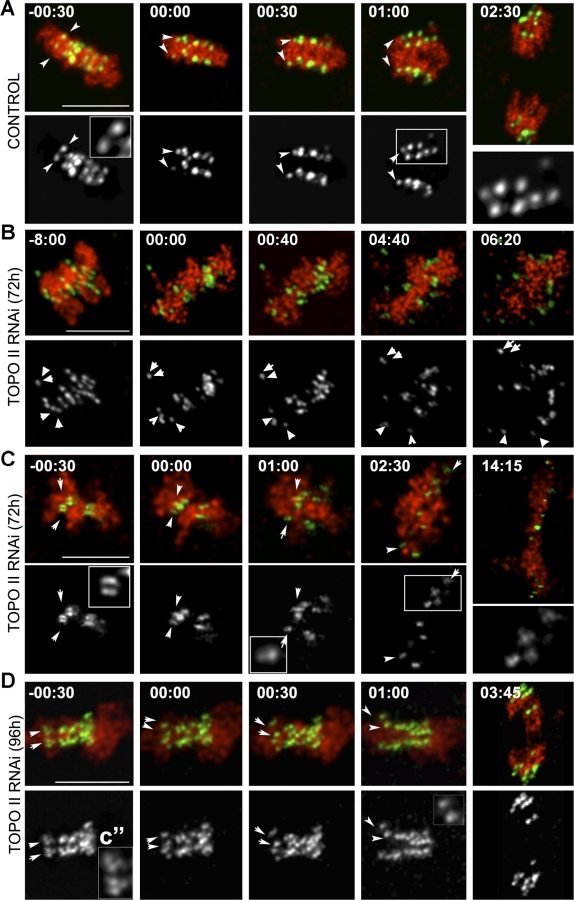
In Vivo Analysis of Chromosome Segregation during Mitosis in TOPO II–Depleted Cells Still images from videos of S2 cells progressing through mitosis after depletion of TOPO II are shown. *Z*-stacks were collected in both (A) control and (B–D) TOPO II–depleted S2 cells stably expressing the centromere marker CID-GFP and histone RFP-H2B. For TOPO II–depleted cells, 16 cells at 72 h and 18 cells at 96 h were recorded. Centromeres were identified by following each pair of CID-GFP dots through every optical layer (*n* = 10; Δ*Z* = 1 μm) at each time point (see Videos S1–S4). (A) In control cells, the centromere (green and individual channel below) is seen as two dots (white arrows) on each side of the chromosomes (red) at metaphase plate (−00:30). Higher magnification of a centromere CID-GFP pair is also shown. After anaphase onset (00:00), centromeres separate and segregate to opposite poles as individual dots (white arrows). Magnification of the white inset (01:00) showing separated CID centromere marker during anaphase is shown on the right. (B) After depletion of TOPO II at 72 h, chromosomes fail to align properly on the metaphase plate when compared to controls. A centromere, visualized as two CID-GFP pairs, that does not align to the metaphase plate can also be observed (white arrows on the top). After anaphase onset (00:00), chromatin masses are observed and a kinetochore pair is clearly observed as migrating to the same pole (bottom, white arrows). (C) At 72 h after the addition of RNAi, extensive chromatin bridges are observed after anaphase onset (00:00), although some centromere pairs (white arrows) can also be detected (white insets). (D) After depletion of TOPO II at 96 h, chromosomes appear to exhibit congression to the metaphase plate (−0:30). Higher magnification insets (CID signal below) show these to consist of paired dots (white arrows). At anaphase onset (00:00), two chromatin masses begin to segregate to opposite poles, but centromere pairs fail to disjoin. See inset of higher magnification of the centromere pairs at time 01:00. Scale bar represents 5 μm.

To directly assess whether TOPO II function is required at centromeres, we depleted the single TOPO II isoform from *Drosophila* S2 cells stably expressing fluorescent markers for chromatin (mRFP-H2B) and centromeres (CID-GFP) [24] by RNAi treatment (Figure 2 and Videos S1–S4). In order to visualize individual sister centromeres at high temporal and spatial resolution, TOPO II–depleted cells were imaged by four-dimensional (4D) time-lapse fluorescence microscopy in which the distance between the optical layers of the *Z*-stack was kept to less than 1 μm. In control cells, chromosomes congression occurs normally, and as anaphase starts, CID-GFP pairs disjoin and move poleward (Figure 2A and Video S1). However, in TOPO II–depleted cells, while chromosomes appear to exhibit normal congression, centromeres of sister chromatids remain on the same side of the metaphase plate, fail to disjoin, and move towards the same pole during anaphase (Figure 2B and 2C and Video S2). Nevertheless, in many cells after 72 h of RNAi treatment, chromatin bridges presumably linking chromosome arms are clearly observed (Figure 2C and Video S3). At later times after RNAi treatment (96 h), most cells show chromosome nondisjunction (Figure 2D and Video S4). Noteworthy, most of the analysis after TOPO II depletion was carried out in cells that did not show extensive polyploidy, as ascertained by chromosome and centromere labeling, indicating that they had not undergone multiple cell cycles. After long RNAi treatment, a small proportion of polyploid cells were observed (Figure S1). These cells are characterized by the presence of chromosomes that are attached by their nondisjoined centromeres, as would be expected if in the previous cycles, proper centromere separation failed. This effect is apparently not due to a failure to replicate centromeric DNA as shown by Southern blotting analysis (Figure S1).

### In the Absence of TOPO II, Chromosomes Show Syntelic Kinetochore Attachments

**Figure 3:**
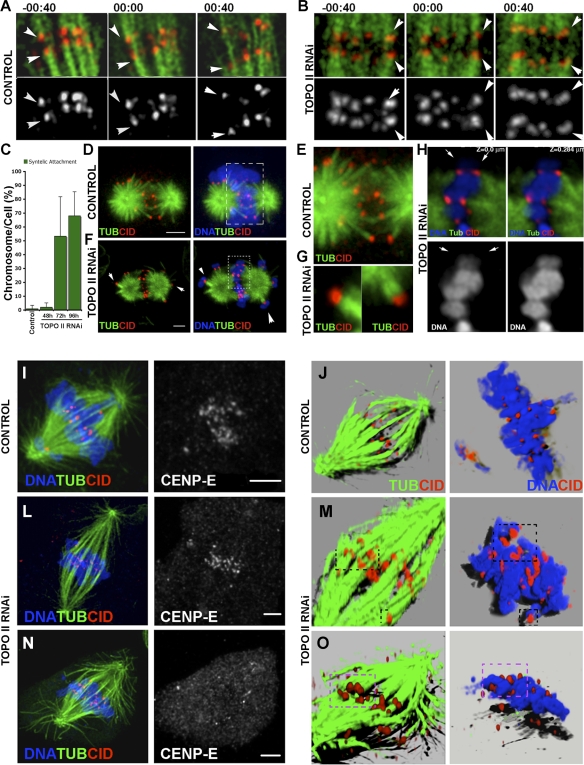
Characterization of Microtubule–Kinetochore Attachment in TOPO II–Depleted Cells Selected frames from time-lapse videos of S2 cells stably expressing GFP-α-tubulin (Tub; green) and CID-mCherry (red and individual channel below) in (A) control or (B) TOPO II–depleted cells are shown. Each *Z*-stack with 10 μm was collected every 40 s. Anaphase onset is set as time 00:00 (see Videos S5 and S6). (A) In control cells (*n* = 15), each CID-labeled centromere binds individual microtubules bundles from opposite spindle poles, demonstrating an amphitelic chromosome attachment (white arrows). (B) In TOPO II–depleted cells (*n* = 18), CID-mCherry is seen on the same side of the metaphase plate (white arrows) interacting with microtubule bundles from the same pole in a clear syntelic attachment (−00:40). After anaphase onset (00:00–00:40), centromeres segregate together and fail to disjoin. (C) Quantification of syntelic attachments observed in vivo during prometaphase and metaphase both in control and TOPO II–depleted cells. More then 53% of the chromosomes were syntelically bound to the microtubules 72 h (*n* = 12) after the beginning of the RNAi treatment, and this slightly increased at 96 h (67%, *n* = 10). Analysis of microtubule–kinetochore interaction in (D and E) control or (F–H) TOPO II–depleted cells. To analyze microtubule–kinetochore interaction directly at metaphase, cells were subjected to the MG132-Taxol assay [25]. Cells were immunostained for CID (red) to identify the centromere, for α-tubulin (green), and for DNA (blue). (D) In control cells, CID staining always appeared as two dots on every chromosome, which show mostly amphitelic attachment. (E) Higher amplification of dashed box in (D). (F) In TOPO II–depleted cells, chromosomes are not confined to the central region between the two asters but are also observed at the periphery, suggesting syntelic chromosome attachment (arrowheads). (G) Higher magnification of the chromosomes indicated by the arrowheads. (H) Higher magnification of two optical sections from the image shown in the dashed box in (F). Note that chromosomes are paired at the metaphase plate (white arrows and individual channel below). (I–O) Immunolocalization of CID (red), α-tubulin (green), DNA (blue), and the kinesin CENP-E (separate channel) in asynchronous (I) control or (L and N) TOPO II–depleted cells. Similar to control cells, after depletion of TOPO II, CENP-E localizes properly to the kinetochores early, but not at anaphase onset. Rendering of these *Z*-stacks reveals that although CID staining is observed in (J) control cells as organized pairs of dots at the metaphase plate, in the absence (M and O) of TOPO II, CID staining is visible as larger dots over the chromatin. Interestingly, in control cells, single dots interact with individual microtubule bundles ([J] left panel), unlike in TOPO II–depleted cells in which microtubules are binding CID-labeled centromere pairs ([M and O] left panel). Some of these unresolved centromeres seen on chromosomes (right panel) in (M and O) are highlighted with a dashed black box and are also shown on the left panel. (O) In TOPO II–depleted cells, when CENP-E is no longer associated with kinetochores, indicating anaphase onset, CID centromere markers are observed as paired dots at each side of the metaphase plate. Rendering of the *Z*-stack visibly shows paired dots on the chromatin ([O] right panel). Scale bar represents 5 μm.

In order to determine why sister centromeres fail to disjoin after TOPO II depletion, cells stably expressing GFP-α-tubulin and CID-mCherry to specifically label spindle microtubules and centromeres were treated with RNAi, and mitotic progression was followed by time-lapse fluorescence microscopy (Figure 3A and 3B and Videos S5 and S6). As expected, in control cells, chromosomes show mostly amphitelic attachments, congression occurs normally, and bundles of spindle microtubules are easily observed associated with individual kinetochores (Figure 3A and Video S5). However, 72 h after TOPO II RNAi, most chromosomes show syntelic attachment and are oriented towards the same spindle pole (Figure 3B and 3C and Video S6). To confirm these observations, we performed an assay designed to quantify the nature of microtubule–kinetochore interactions in S2 cells [25]. For this assay, cells were arrested in mitosis with the proteasome inhibitor MG132 and subjected to a high dose of Taxol, which over a short period of time causes the collapse of the bipolar spindle into a monopolar configuration. This monopolar structure now contains the chromosomes distributed at the periphery of the aster, and microtubule–kinetochore interactions can be easily scored (Figure S2). We find that in control and TOPO II–depleted cells, more than 95% of the chromosomes had both kinetochores attached to microtubules in a syntelic configuration soon after significant depletion of TOPO II occurs. If these cells are analyzed when the two asters are in the process of collapsing, it is possible to ascertain whether the attachment is amphitelic, with chromosomes localized between the asters, or syntelic/monotelic, when located at the periphery of the aster (Figure 3D–3H). In control cells (*n* = 24), most chromosomes (78%) exhibited a clear amphitelic configuration, whereas in TOPO II–depleted cells (*n* = 20), most chromosomes (65%) localized at the periphery of the two asters with a clear syntelic configuration (Figure 3F and 3G). Consistently, CID fluorescence intensity for the centromere marker CID in TOPO II–depleted cells is almost double that of control cells (unpublished data), and intercentromere distances never increase during mitotic progression (Figure S3). Analysis of microtubule–kinetochore interactions after TOPO II depletion was also performed in asynchronous cells. For this, either control or TOPO II–depleted cells were fixed and stained for CID, α-tubulin, and CENP-meta, the *Drosophila* CENP-E homolog, a kinetochore motor protein whose levels decrease significantly at kinetochores during anaphase [26] (Figure 3I–3O). As expected, we find that CENP-E is present at kinetochores of control and TOPO II–depleted cells in prometaphase (Figure 3I and 3L), but when chromosomes move poleward, CENP-E is undetectable (Figure 3N), indicating that all kinetochores are attached to spindle microtubules. Rendering these images for the staining of CID and tubulin confirms that in the absence of TOPO II, kinetochore bundles are associated with pairs of CID dots, unlike control cells in which they associate with single CID dots (Figure 3J, 3M, and 3O). Taken together, these results indicate that in the absence of TOPO II, sister centromeres fail to disjoin and chromosomes show mostly syntelic microtubule attachments.

### TOPO II Activity Is Required to Establish Amphitelic Kinetochore Attachment

**Figure 4:**
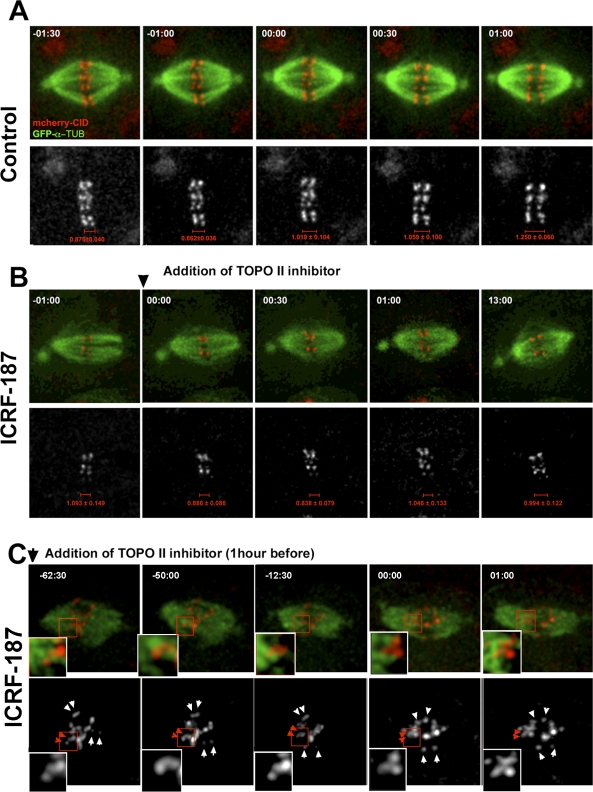
In Vivo Analysis of Centromere Segregation in S2 cells after Incubation with TOPO II Inhibitors Selected stills from time-lapse videos of S2 cells stably expressing GFP-α-tubulin (GFP-α-TUB; green) and CID-mCherry (red and white individual channel below) in (A) control or (B and C) ICRF-187–treated cells (see Videos S7–S9). Each *Z*-stack with 10–12 μm was collected every 30 s. (A) In control cells (*n* = 15), each CID-labeled centromere binds individual microtubule bundles from opposite spindle poles with a clear amphitelic attachment till the anaphase onset (00:00). (B) The analysis of TOPO II activity was performed by adding the TOPO II inhibitor ICRF-187 after chromosome congression and alignment of all the centromeres at the metaphase plate. Accordingly, centromeres exhibit a clear amphitelic attachment (−1:00), which is maintained after the addition of ICRF-187 to a final concentration of 50 μg/ml (00:00–13:00). This analysis was performed in 21 different cells. (C) Cells were recorded after 1 h of incubation with 50 μg/ml ICRF-187, and 18 different cells were recorded. Under these conditions, most of the centromeres (red insets and red arrows) exhibit a syntelic configuration until the anaphase onset (00:00). Unlike the kinetochores that are amphitelically bound to the microtubules (white arrows), the syntelic centromeres segregate paired after the anaphase onset.

Long-term inhibition of TOPO II with drugs affects S2 cells during G2 and mitotic entry, resulting in severe abnormalities in chromosome structure that prevented us from using inhibitors to carry out a thorough analysis of the role of TOPO II during mitotic progression. However, we hypothesized that short incubations with the inhibitors might allow us to study its effects in living cells as they enter mitosis. Accordingly, cells were treated for short periods of time with ICRF-187. S2 cells stably expressing GFP-α-tubulin and CID-mCherry were imaged by 4D microscopy during ICRF-187 treatment, before and after establishment of the metaphase plate (Figure 4 and Videos S7–S9). We find that inhibition of TOPO II activity after chromosomes have reached the metaphase plate and established bipolar attachment does not have an effect on the kinetochore–microtubule interaction (Figure 4A and 4B). However, if the TOPO II inhibitor is added before nuclear envelope breakdown, as inferred by the exclusion of GFP-α-tubulin from the nucleus (unpublished data), during prometaphase, we observe that 62.5% of chromosomes/cell (*n* = 12) exhibit syntelic chromosome attachments, with kinetochore pairs moving poleward without separating their centromeres (Figure 4C). These observations not only confirm our live-cell analysis of TOPO II–depleted cells after RNAi, but also indicate that TOPO II activity at early stages of mitosis plays an important role at centromeres to promote normal chromosome biorientation. However, once amphitelic attachments are achieved, TOPO II activity is not required for their maintenance.

### TOPO II Is Required for Centromere Separation Independently of the Cohesin Complex

**Figure 5:**
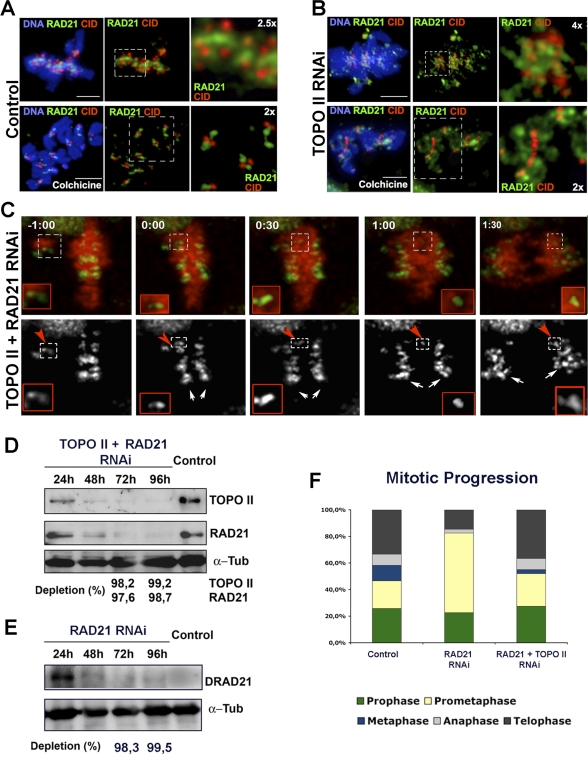
Activity of the Cohesin Complex during Mitotic Progression of TOPO II–Depleted Cells Immunolocalization of RAD21 (green), a subunit of the cohesin complex, CID (red), and DNA (blue) in (A) control or (B) TOPO II–depleted S2 cells. (A) In control cells, cohesin subunit RAD21 localizes between sister chromatids during prometaphase and metaphase and is mostly confined to the centromere in colchicine-treated cells (see higher magnifications of the dashed boxes on the right). (B) However, in TOPO II–depleted cells, RAD21 is seen along the chromatin and not just confined to the centromere region (see higher magnifications of the dashed boxes on the right). This difference is particularly evident after colchicine treatment (panel below) where cohesin staining spreads well beyond the centromere. (C) Images from videos of S2 cells stably expressing the centromere marker CID-GFP (green and individual channel below) and the histone RFP-H2B (red) depleted for both RAD21 and TOPO II. For codepletion experiments, 12 cells were recorded and *Z* stacks were collected every 15 s. Centromeres were identified at every optical section (*n* = 10; Δ*Z* = 1 μm) (see Videos S10 and S11). The images show (−1:00 and 0:00) two pairs of chromosomes exhibiting congression to the metaphase plate (red arrowheads and higher magnification of white dashed boxes in red boxes below), but as the cell exits mitosis (0:30–1:30), sister chromatids fail to separate, and segregate to the pole together. (D and E) Western blot analysis of S2 cells treated with dsRNAi to (D) codeplete RAD21 and TOPO II or (E) deplete DRAD21 only. Note that both proteins were significantly depleted after 72 h of treatment. (F) Progression through mitosis was determined by immunostaining to detect cyclin B. After depletion of DRAD21, the percentage of cells in prometaphase is significantly higher when compared to controls, suggesting problems in chromosome alignment. Interestingly, after codepletion of TOPO II and RAD21, the mitotic profile is similar to control, and normal levels of cells in prometaphase or metaphase are observed. Scale bar represents 5 μm.

Previously, it has been demonstrated that as cells progress through late prometaphase and metaphase, cohesin is removed from the chromosome arms, remaining only at the centromere until the metaphase–anaphase transition [27–29]. Therefore, it remains possible that sister centromeres fail to disjoin after depletion of TOPO II due to an inappropriate accumulation of cohesin. In order to test this hypothesis, we determined the localization of the cohesin subunit RAD21/SCC1 in control and TOPO II–depleted cells before and after incubation with colchicine. In control cells at metaphase or after colchicine incubation, cohesin localizes as a clearly defined stripe between centromeres (Figure 5A), but after depletion of TOPO II, cohesin shows a very abnormal distribution, which extends into the chromosome arms even after mitotic arrest (Figure 5B). To test whether the inappropriate localization of cohesin accounts for the observed centromere nondisjunction in TOPO II–depleted cells, we performed simultaneous depletion of TOPO II and RAD21 by RNAi and followed mitotic progression and sister chromatid segregation (Figure 5C). Although both proteins were efficiently depleted, cells progressed through mitosis, showing lagging chromatids or chromosomes (Figure S4). The 4D microscopy studies of mitotic cells stably expressing CID-GFP and RFP-H2B after simultaneous depletion of TOPO II and RAD21 show that the behavior of sister chromatids is identical to that of cells depleted of TOPO II alone and very different from RAD21 RNAi (Figure 5F and Videos S10 and S11). Cells depleted of RAD21 enter mitosis with separated sister chromatids and arrest in a prometaphase-like state (Figure S5, and Video S12) because they fail to inactivate the SAC [30,31]. However, in cells depleted of both TOPO II and RAD21, closely paired sister chromatids reach the metaphase plate and, during anaphase, fail to disjoin, segregating together to the same spindle pole. These results demonstrate that inappropriate localization of RAD21 is unlikely to be responsible for the centromere nondisjunction phenotype observed after depletion of TOPO II.

### TOPO II–Depleted Cells Are Able to Satisfy the Spindle Assembly Checkpoint

**Figure 6:**
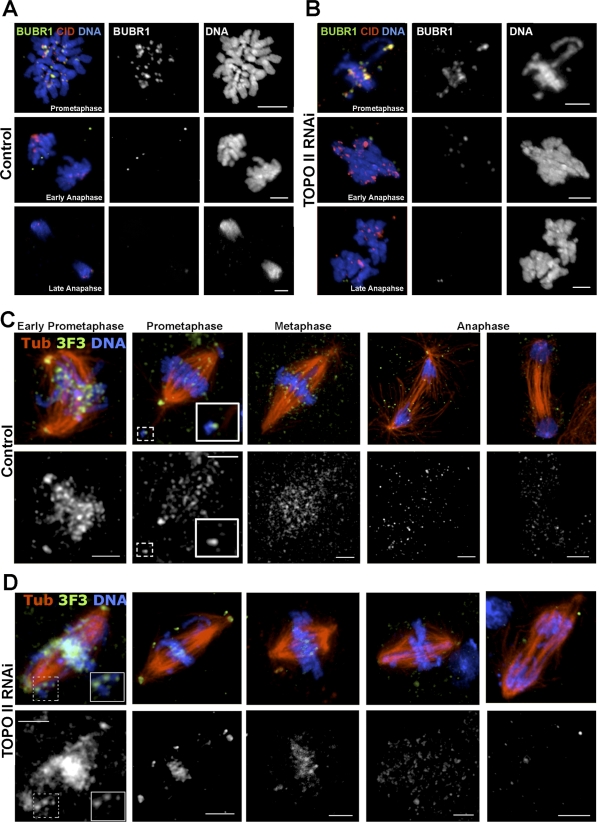
Spindle Assembly Checkpoint Activity during Mitotic Exit of TOPO II–Depleted Cells (A) Control and (B) TOPO II–depleted cells show kinetochore labeling of BubR1 in prometaphase and metaphase, whereas after anaphase onset, BubR1 is barely detectable. These results suggest that after depletion of TOPO II, SAC protein shows a pattern of accumulation at kinetochores that is indistinguishable from controls. The 3F3/2 phosphoepitope immunolocalization in (C) control or (D) TOPO II–depleted cells. Cells were immunostained for α-tubulin (red), 3F3/2 (green and separated channel below), and counterstained for DNA (blue). (C) During early prometaphase, 3F3/2 centromere staining is intense, but in late prometaphase, the intensity decreases significantly except in kinetochores of misaligned chromosomes (see boxes). After anaphase onset, 3F3/2 staining is absent. (D) In TOPO II–depleted cells, 3F3/2 staining localizes over the chromosomes in early prometaphase although its localization can also be observed as discrete dots at the kinetochores of misaligned chromosomes at the poles (see boxes). The 3F3/2 signal at the kinetochores is reduced during prometaphase–metaphase and is absent in anaphase similarly to controls. Scale bar represents 5 μm.

Together with the data, our results strongly suggest that depletion of TOPO II causes the formation of a physical linkage between sister centromeres, likely provided by DNA concatameres, which is not resolved during the metaphase–anaphase transition. As a consequence, TOPO II–depleted cells segregate entire chromosomes rather than sister chromatids. Apparently, this is not due to the inability of TOPO II–depleted cells to satisfy the SAC since they showed no significant mitotic delay and anaphase-promoting complex (APC/C)-dependent proteolysis of RAD21 and cyclin B occurs (Figure S6). If depletion of TOPO II causes failure to resolve sister chromatids and syntelic attachments, how then do these cells satisfy the SAC? To address this question, we first determined the localization of SAC proteins after TOPO II RNAi. We find that in prometaphase, BubR1 accumulates strongly at kinetochores, while during anaphase, the level of BubR1 was significantly reduced, suggesting that TOPO II–depleted cells are able to inactivate the SAC, just like control cells (Figure 6A and 6B). To further determine whether syntelic chromosomes undergo proper tension during mitosis, TOPO II–depleted cells were immunostained with the 3F3/2 monoclonal antibody that specifically detects kinetochore phosphoepitopes in the absence of tension [32–34]. We find that control and TOPO II–depleted cells behave very similarly so that 3F3/2 kinetochore phosphoepitopes are strongly labeled in prometaphase, become significantly reduced during prometaphase/metaphase, and are undetected in anaphase (Figure 6C and 6D). These results indicate that SAC satisfaction in TOPO II–depleted cells with syntelic attachments correlates with the dephosphorylation of 3F3/2 epitopes but does not require extensive interkinetochore stretching.

### TOPO II–Depleted Cells Show Mislocalization of Chromosomal Passenger Proteins

**Figure 7:**
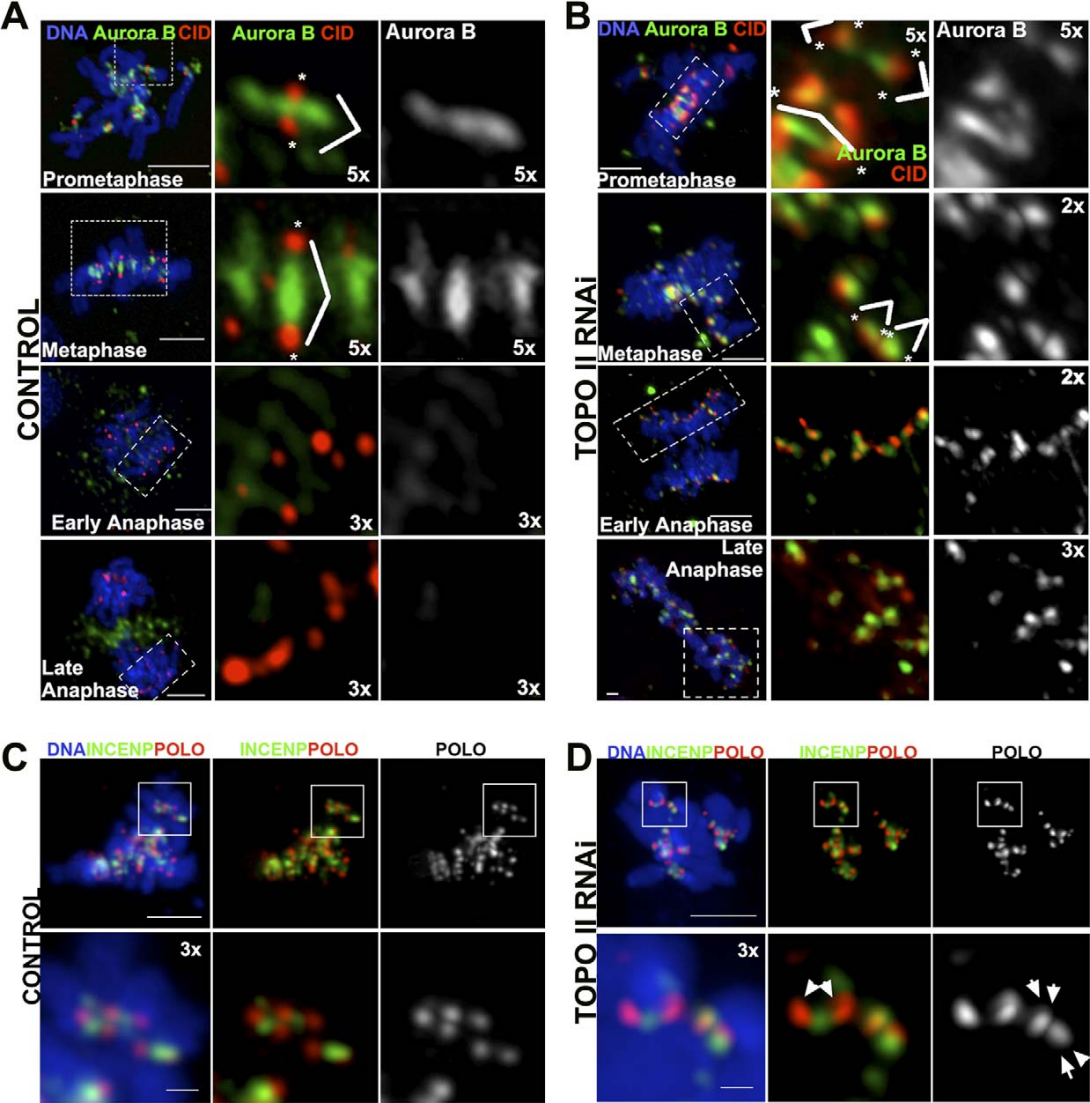
Localization of Chromosome Passenger Proteins during Mitotic Progression after Depletion of TOPO II Aurora B (green), CID (red), and DNA (blue) in (A) control or (B) TOPO II–depleted cells were localized by indirect immunofluorescence. (A) In control cells, Aurora B localizes between sister chromatids during prometaphase and metaphase. At anaphase, Aurora B localizes to the central spindle and is no longer observed between the sisters at the primary constriction, and at late anaphase, localizes to the spindle mid-zone. Magnifications of the images from dashed boxes are indicated. Individual CID dots are also shown, indicated with asterisks (*), and pairs of CID are identified by white lines. (B) In TOPO II–depleted cells, Aurora B is found between the two CID signals in prometaphase. At metaphase, the staining almost colocalizes with the centromere, remaining closely associated throughout anaphase, and there is no transfer of Aurora B to the central spindle. In late anaphase, staining remains always tightly associated with all centromeres. Magnifications of the images from dashed boxes are indicated. Individual CID dots are also shown, indicated by asterisks (*), and pairs of CID are identified by white lines. Immunolocalization of INCENP (green), POLO (red), and DNA (blue) in (C) control or (D) TOPO II–depleted cells. (C) In control cells, INCENP always localizes between sister kinetochores during prometaphase (white insets and magnification below). (D) In TOPO II–depleted cells, INCENP is found either between the kinetochore pairs (white insets and magnification below) or associated with the kinetochore in prometaphase (white arrows in the POLO channel). Scale bar represents 5 μm.

Previously, it has been shown that the correction of improper microtubule–kinetochore attachments requires Aurora B kinase activity as part of a tension-sensing mechanism on centromeres [1]. Aurora B is part of the chromosomal passenger complex (CPC) that localizes to the inner centromere during prometaphase/metaphase and transfers to the spindle midzone at anaphase onset and the mid body in telophase [35]. Therefore, we analyzed whether the localization of CPC proteins after TOPO II depletion was compromised and could be responsible for the inability of these cells to release syntelic attachments (Figure 7). In control cells, Aurora B localizes to the inner centromere region during prometaphase/metaphase and is transferred to the spindle midzone when cells initiate anaphase [36] (Figure 7A). However, in TOPO II–depleted cells Aurora B localization is abnormal (Figure 7B). In prometaphase, Aurora B remains associated with sister kinetochores, does not stretch across the metaphase plate, remains associated with inner centromeres of syntelic chromosomes during anaphase, and fails to transfer to the spindle midzone. A very similar abnormal pattern of localization was also observed for INCENP, a member of the CPC, which regulates Aurora B activity (Figure 7C and 7D). These observations indicate that TOPO II is essential for the organization of the inner centromere so that the CPC can show a normal pattern of localization during mitosis.

### Depletion of TOPO II Causes a Significant Reduction in Aurora B Kinase Activity

**Figure 8:**
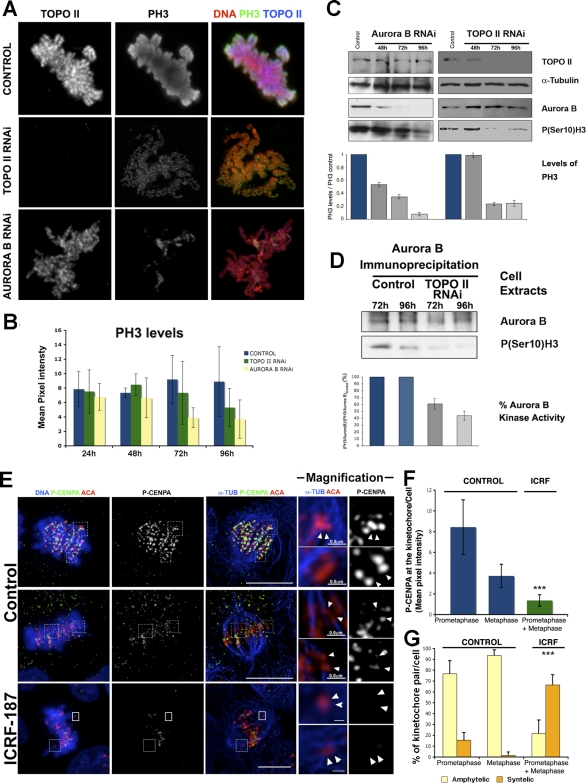
Analysis of Aurora B Kinase Activity after Depletion or Inhibition of TOPO II Activity in S2 and HeLa Cells (A) Immunofluorescence analysis for histone H3 Ser10 phosphorylation (PH3) in control, TOPO II–, and Aurora B–depleted cells. In control cells, both TOPO II and PH3 localize throughout chromosomes. In TOPO II–depleted cells, PH3 levels are significantly lower than in control. More significantly, in Aurora B–depleted cells, PH3 levels are even lower than in the TOPO II–depleted cells. (B) Quantification of the mean pixel intensity for PH3 signals in control, TOPO II–, and Aurora B–depleted S2 cells every 24 h at each time point of the experiment. Differences between the control and the samples of the two RNAis start to be observed at 72 h of depletion. Error bars indicate the standard deviation of the average. Compared to the control cells, the differences in levels of PH3 96 h after depletion of TOPO II (59.1%) or Aurora B (62,1%) are significant (*p* < 0.005) and (*p* < 0.001), respectively. (C) Depletion of TOPO II and Aurora B was also monitored by western blot. Both TOPO II and Aurora B protein were barely detectable 72 h (>80% reduction) after the addition of dsRNAs. In Aurora B–depleted cells, the reduction of Aurora B levels parallels the reduction in the levels of PH3 reactivity. Note that after depletion of TOPO II, the level of Aurora B is not affected, but PH3 levels are reduced. Below, the quantitative analysis of PH3 reactivity is shown. The values represent the ratio of total pixel intensity of PH3 band to that of α-tubulin. (D) Aurora B kinase assay was performed with immunoprecipitated Aurora B from control and TOPO II–depleted extracts (72 and 96 h). Kinase activity was measured by quantification of histone H3 phosphorylation normalized for the amount of Aurora B immunoprecipitated in each assay. Below, the quantification of PH3 reactivity from the in vitro kinase assay is shown. Note the severe reduction in Aurora B kinase activity after 96 h of TOPO II depletion. (E) Immunolocalization of phospho-CENPA (P-CENPA; green and white separated channel), anticentromere antibody (ACA; red) and DNA or α-tubulin (blue) in control HeLa and ICRF-187–treated HeLa cells. In control cells, phospho-CENPA localizes strongly to the (white arrows) unaligned kinetochores (upper figure and insets). In metaphase, the chromosomes are amphitelically attached to the microtubules (white arrows), and in agreement, they exhibit lower levels of phospho-CENPA at the kinetochores (middle and magnifications of boxes). HeLa cells after TOPO II depletion consistently show lower levels of phospho-CENP-A at their kinetochores. The kinetochores that are syntelically bound to the microtubules (white arrows) (bottom and magnifications) do not stain for phospho-CENPA. (F) Quantification of phospho-Ser(7)CENPA mean pixel intensity at each kinetochore pair/cell in control and in ICRF-187–treated HeLa cells. In control cells, the P-CENPA intensity at the kinetochore is consistently higher in prometaphase (8.43, *n* = 10 cells) than in metaphase (3.28, *n* = 14). In ICRF-187–treated HeLa cells, P-CENPA levels quantified are more reduced (1.36, *n* = 16). The differences in P-CENPA levels between controls cells and cells treated with TOPO II inhibitor are highly significant (triple asterisks [***] indicate *p* < 0.001). (G) Quantification of amphitelic and syntelic chromosomes per cell both in control and ICRF-187–treated HeLa cells. In control HeLa cells, chromosomes preferentially bind amphitelically to microtubules both in prometaphase (76.7%, *n* = 14; undetermined 7.9%) and in metaphase (93.5%, *n* = 10; undetermined 5.0%). However, in ICRF-187–treated cells, kinetochore pairs interact with microtubules mainly syntelically (66.3%, *n* = 26; undetermined 12.1%), and only 21.5% are found with a bipolar attachment to microtubules. The differences observed in the type of kinetochore–microtubule interactions between ICRF-187–treated HeLa cells and control cells both in prometaphase and metaphase are significant (triple asterisks [***] indicate *p* < 0.001). Scale bar represents 5 μm.

Previous studies in human cells have shown that chromosomes with syntelic attachments experience significant distortion of their centromeres and are not easily identified by the SAC, and that these errors are enhanced when aurora B kinase activity is inhibited [37]. Moreover, it has been shown that Aurora B is normally enriched at sites associated with erroneous microtubule attachments where it promotes microtubule depolymerization [38]. Since in TOPO II–depleted cells Aurora B remains associated with syntelic attachments throughout mitosis, it would be expected that these erroneous attachments would be corrected, unless Aurora B activity is compromised due to structural changes resulting from TOPO II depletion. To test this hypothesis, we analyzed the levels of phosphorylation of histone H3 at Ser 10 (PH3), a known Aurora B substrate [39] (Figure 8A). Immunofluorescence analysis revealed that after TOPO II depletion at 96 h, PH3 levels were reduced almost by half (41%) when compared with control cells (Figure 8B) and not much different from the reduction (62%) observed after RNAi depletion of Aurora B (Figure 8A and 8B). Similar results were obtained after treatment of cells with the TOPO II inhibitor ICRF-187 (Figure S7). Western blot of total protein extracts of TOPO II– and Aurora B–depleted cells confirmed that PH3 levels were significantly reduced (Figure 8C). However, although TOPO II–depleted extracts show a significant reduction in PH3 reactivity, the total Aurora B levels appear unaffected, suggesting that depletion of TOPO II specifically affects the kinase activity of Aurora B. To directly address this possibility, Aurora B was immunoprecipitated from total protein extracts from control or TOPO II–depleted cells and its kinase activity tested in vitro with unphosphorylated histone H3 (Figure 8D). We find that phosphorylation of histone H3 is reduced by half relative to controls when Aurora B is immunoprecipitated from TOPO II–depleted cells. This indicates that either directly or indirectly, TOPO II is required to promote Aurora B kinase activity. To further analyze how TOPO II regulates Aurora B activity at centromeres, we turned to HeLa cells. Cells were treated with the TOPO II inhibitor ICRF-187, and Aurora B kinase activity was quantified by measuring the phosphorylation of Ser7 of the centromeric protein CENP-A that has been found to be a direct substrate of Aurora B [40] (Figure 8E and 8F). In control cells, we find that P-Ser7CENP-A immunoreactivity is very high in most kinetochores during prometaphase, revealing the normal activity of Aurora B at this stage of mitosis. As cells reach metaphase and kinetochore–microtubule interactions become stabilized, P-Ser7CENP-A immunoreactivity is significantly reduced, suggesting that Aurora B kinase activity is normally down regulated at this stage. However, after inhibition of TOPO II, we find that cells in prometaphase display a significant reduction in the level of P-Ser7CENP-A immunoreactivity (Figure 8E–8G), and more than 60% of chromosomes per cell (*n* = 26) display syntelic attachment. These results indicate that TOPO II is also required to establish amphitelic attachment in HeLa cells, similar to what we observed for *Drosophila*, and further demonstrate that TOPO II activity regulates Aurora B kinase activity on chromosomes and more specifically at centromeres.

### Codepletion of BubR1 and TOPO II Restores Histone H3 Phosphorylation and Releases Syntelic Attachments

**Figure 9:**
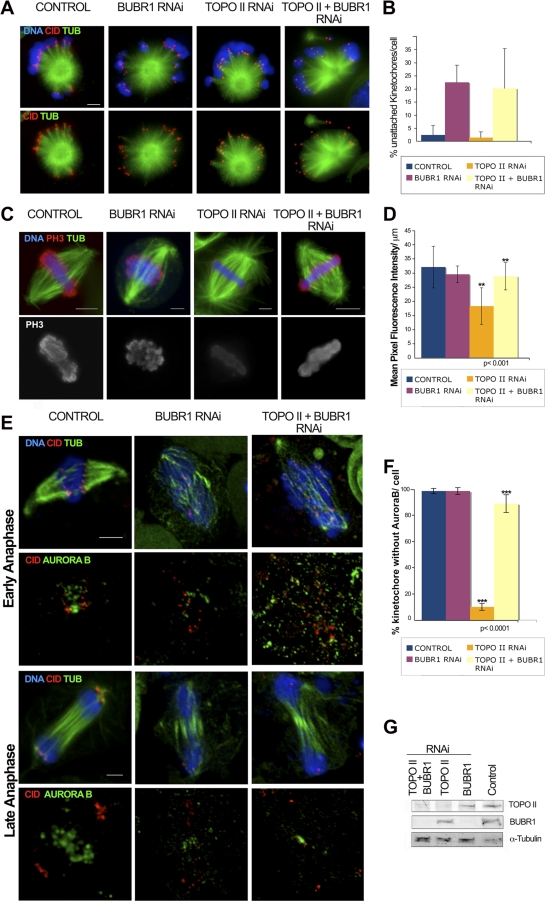
Analysis of Kinetochore Attachment and Aurora B Activity after Simultaneous Depletion of BubR1 and TOPO II (A) Immunofluorescence for α-tubulin (TUB; green), CID (red), and DNA (blue) in control, TOPO II–, BubR1-, and TOPO II/BubR1-depleted cells subjected to the Taxol-MG132 assay. (B) Quantification of kinetochore–microtubule attachment shows that in cells depleted for both TOPO II and BubR1 (*n* = 28), the percentage of unattached kinetochores (20.2%) is significantly higher compared to control (≤2.5%, *n* = 22) or TOPO II–depleted cells (≤1.5%, *n* = 24) (*p* < 0.001) and very similar to the percentage of unattached kinetochores in the absence of BubR1 (22.4%, *n* = 21). The error bars indicate the standard deviation of the average. (C) Immunofluorescence analysis for histone H3 Ser10 phosphorylation (PH3; red), α-tubulin (green) in control, TOPO II–, BubR1-, and TOPO II/BubR1-depleted cells. (D) Quantification of the mean pixel intensity for PH3 signals in control (*n* = 22), BubR1- (*n* = 23), TOPO II– (*n* = 24), and TOPO II/BubR1-depleted (*n* = 26) cells. The reduction of PH3 levels detected in TOPO II–depleted cells compared to control (57%) and to BubR1-depleted cells is partially abolished in TOPO II + BubR1–depleted cells. The PH3 levels are recovered, and the differences quantified between TOPO II and double-depleted cells are significant (*p* < 0.001). Scale bar represents 5 μm. (E) Immunofluorescence of Aurora B (green), CID (red), and DNA in control, TOPO II–, BubR1-, and TOPO II/BubR1-depleted cells. In control cells, as in BubR1- and TOPO II/BubR1-depleted cells, Aurora B does not localize to the centromeres after anaphase onset. (F) Quantification of the number of kinetochores labeled with Aurora B after anaphase onset in control (*n* = 130), BubR1- (*n* = 165), TOPO II– (*n* = 122), and TOPO II/BubR1-depleted (*n* = 189) cells. The association of Aurora B with the kinetochores quantified in TOPO II–depleted chromosomes during anaphase (≥90%) is abolished in double-depleted cells for TOPO II and BubR1 (11.13%). The difference between TOPO II– and TOPO II/BubR1-depleted cells is significant (*p* < 0.0001). (G) Western blot of the total protein cell extracts was used to monitor RNAi depletion.

The observations described above demonstrate that TOPO II activity at the centromere is required for the normal function of Aurora B. However, these studies do not distinguish whether TOPO II controls Aurora B kinase activity directly or indirectly. Previous studies showed that inhibition of Aurora kinase activity suppresses the misalignment/attachment defects in BubR1-depleted cells [41]. This effect was shown to be due to an increase in Aurora B kinase activity after BubR1 depletion [41]. Similarly, small interfering RNAi (siRNAi) depletion of Aurora B in cells where BubR1 was also knocked down, results in more stable kinetochore attachment [42]. Therefore, given that in the absence of TOPO II, sister centromeres appear unable to resolve, bind microtubules syntelically, and segregate to the same pole, we tested whether BubR1 might be responsible for negatively regulating Aurora B activity in these cells (Figure 9). To address this issue, we measured microtubule–kinetochore attachment using the Taxol-MG132 assay, as well as mitotic PH3 reactivity in control, BubR1-, TOPO II–, and TOPO II/BubR1–depleted S2 cells. Single or double RNAi treatments were carried out and the respective protein levels quantified by western blotting (Figure 9G). As described before, the Taxol-MG132 assay shows that in control or TOPO II–depleted cells, most chromosomes are attached to spindle microtubules (Figure 3), but when BubR1 is depleted alone, many cells show either unattached or mono-oriented chromosomes [25] (Figure 9A and 9B). Interestingly, when BubR1 and TOPO II are simultaneously depleted, we observed a large increase in the number of unattached kinetochores (Figure 9A and 9B). This result indicates that removing BubR1 from TOPO II–depleted cells can reactivate the correction mechanism and allow the release of syntelic attachments. To determine whether this was the result of Aurora B kinase activity, we then analyzed PH3 levels by immunofluorescence microscopy. The results show that depletion of BubR1 in the absence of TOPO II is able to restore normal PH3 levels on chromatin, suggesting that Aurora B kinase is now active (Figure 9C and 9D). We also analyzed the localization of Aurora B during mitotic exit of cells after simultaneous depletion of TOPO II and BubR1 (Figure 9E). Interestingly, during early anaphase, Aurora B is found tightly associated with the centromeres but in late anaphase is no longer detected and accumulates in the spindle midzone (Figure 9E and 9F). These results suggest that TOPO II is unlikely to have a direct role in regulating the kinase activity of Aurora B. Instead, the abnormal configuration of the centromere resulting from TOPO II depletion appears to cause inappropriate inhibition of Aurora B through BubR1.

## Discussion

Our live-cell studies show that TOPO II has a central role in promoting structural changes of the centromeric DNA that are essential for their individualization and separation at metaphase–anaphase transition. This process is clearly independent of the cohesin complex since depletion of RAD21 causes a SAC-dependent prometaphase-like arrest with separated sister chromatids [4,43], which can be overcome by simultaneous depletion of condensin [4] or TOPO II (Figure 5). Therefore, whereas the role of cohesin degradation in defining the initial steps of sister chromatid separation is well established, it is clear that these events must be tightly coordinated with TOPO II activity. Although it was previously suggested that TOPO II might have a role at the centromere [15,44], previous functional studies have failed to detect any effect on centromere separation during mitotic exit [12,31,45]. Therefore, our results provide the first direct evidence that TOPO II activity is required for centromere disjunction during mitosis.

Our results further show that the structural changes of centromeric DNA resulting from the decatenation activity of TOPO II appear to be essential for the establishment of amphitelic microtubule–kinetochore attachments. In the absence of TOPO II, the SAC appears unable to detect sister kinetochores that are attached to the same pole. One possible explanation is that in TOPO II–depleted cells, Aurora B kinase activity is down-regulated, and given its role in activating the SAC in response to loss of tension, cells cannot respond properly and therefore do not activate the correction mechanism. Interestingly, it has been shown that during exit from meiosis I, when sister chromatids do not disjoin, Aurora B and INCENP remain at the inner centromere [46], similar to what we observe after depletion of TOPO II. Thus, chromosome segregation in TOPO II–depleted cells resembles the first meiotic division when both sister kinetochores are oriented towards the same pole, suggesting that TOPO II may play a role in modulating centromere structure required for proper bivalent biorientation.

The functional interaction between TOPO II and Aurora B has been explored before. In human cells, TOPO II was demonstrated to be an in vitro substrate of Aurora B [47]. Here, we show that depletion of TOPO II causes a down-regulation of Aurora B kinase activity. We observed that the levels of chromosome-associated PH3 staining during prometaphase and metaphase are significantly reduced after depletion of TOPO II but also find that after treatment of S2 cells with a TOPO II inhibitor ICRF-187, which compromises TOPO II activity without changing its chromosomal localization, PH3 reactivity is also significantly reduced. In agreement, inhibition of TOPO II catalytic activity in human cells also results in a dramatic reduction on the phosphorylation levels of Ser 7 CENP-A phosphoepitope, indicating that Aurora B activity is affected not only on chromosomes, but also specifically at centromeres.

The reduction in Aurora B kinase activity could either result from a direct effect of TOPO II or, more likely, through an alteration of the structure of the centromere that occurs as a consequence of TOPO II depletion, and therefore likely represents an indirect effect. We addressed this issue by codepleting BubR1, a SAC protein thought to be involved in inhibiting Aurora B at kinetochores, and find that indeed, codepletion of TOPO II and BubR1 restores normal Aurora B kinase activity and releases syntelic chromosome attachments. Previous work in HeLa cells has shown that either inhibition of Aurora B kinase activity [41] or depletion of Aurora B by RNAi [42] suppresses the misalignment/attachment defects observed in BubR1-depleted cells. In agreement, an increase in Aurora B kinase activity has been reported in the absence of BubR1 [41]. Taken together, our results suggest that BubR1 is able to inhibit Aurora B when in close proximity, so that in early stages of prometaphase, when microtubule attachment is being established and there is not sufficient tension, Aurora B is not activated. However, the increase in tension upon chromosome biorientation, which increases the distance between BubR1 and the centromere during prometaphase, allows Aurora B activation. Indeed, after TOPO II depletion, sister centromeres remain very close, and therefore BubR1 could be responsible for inhibiting Aurora B. Depletion of both BubR1 and TOPO II results in reactivation of Aurora B kinase activity, release of syntelic attachments, and the formation of unattached or mono-oriented chromosomes. These data support recent observations suggesting that activation of microtubule–kinetochore correction mechanisms during mitosis is dependent on centromere plasticity, but not on centromere elasticity [48]. In summary, our observations demonstrate that TOPO II is required for structural changes at the centromere during their resolution, and in turn, this allows normal function of Aurora B, maintenance of SAC activity, and eventual activation of the mechanisms that correct abnormal microtubule–kinetochore attachments.

## Materials and Methods

### Double-stranded RNA interference.

RNAi was performed in *Drosophila* S2 tissue culture cells as previously described [49]. A 1,000-bp EcoRI-HindII and an 800-bp EcoRI-KpnI fragment from the 5′ end of TOPO II (RE49802) and RAD21 cDNAs [4], respectively, were cloned into both pSPT18 and pSPT19 expression vectors (Roche). The recombinant plasmids were used as templates for RNA synthesis using the T7 Megascript kit (Ambion), and 15 μg of double-stranded RNA (dsRNA) were added to 10^6^ cells in all RNAi experiments. At each time point, cells were collected and processed for immunoblotting or immunofluorescence. For immunoblotting, cells were collected by centrifugation, washed in PBS supplemented with protease inhibitors (Roche), and resuspended in 20 μl of SDS sample buffer before loading on a 5%–20% gradient SDS-PAGE. When required, cells were incubated with 30 μM colchicine prior to fixation (Sigma).

### Time-lapse fluorescence imaging.

Live analysis of mitosis was done on S2 cells stably expressing GFP-CID and RFP-H2B [24], as well as in CID-Cherry and GFP-α-tubulin. A cell line stably expressing both GFP-α-tubulin and CID-mCherry was created by transfecting S2 GFP-α-tubulin cells (a kind gift from Gohta Goshima [50]) with pMT_cid_mCherry_BLAST vector (designed from the pMT_cid_gfp, a kind gift from Karpen), pCoBLAST (Invitrogen), and pRSET-B_mCherry (Invitrogen). Control or TOPO II RNAi-treated cells were incubated for 72–96 h and plated on glass coverslips treated with 30 μg/ml concanavalin A (Sigma). Time-lapse images were collected every 20 s for CID-GFP and RFP-H2B and every 45 s for CID-mCherry and GFP-α-tubulin, by Scanning Confocal Microscope Leica SP2 AOBS SE (Leica Microsystems), using the software provided by the manufacturer, Software LCS (Leica Microsystem). Each *Z*-stack is composed of ten images at 0.8–1-μm intervals. Data stacks were deconvolved with the Huygens Essential version 3.0.2p1 (Scientific Volume Imaging). Image sequence analysis and video assembly was done with ImageJ Software (NIH) and Quicktime 7 (Apple Computer).

### Immunofluorescence in *Drosophila* S2 cells.

For immunostaining, 2 × 10^5^ cells were centrifuged onto slides, simultaneously fixed and extracted in 3.7% formaldehyde (Sigma), 0.5% Triton X-100 in PBS for 10 min, and then washed three times for 5 min in PBS-T (PBS with 0.05% Tween 20). Blocking and incubating conditions were performed as described previously [4].For immunofluorescence with the monoclonal antibody (mAb) 3F3/2, cells were grown on glass coverslips, after which they were simultaneously lysed and fixed in lysis/fixation buffer for 2 min (1.5× PHEM, 2% Triton X-100, 0.15% glutaraldehyde, 2% formaldehyde, 10 μM microcystin LR) by 1:1 dilution directly in the culture dishes. Detergent-extracted S2 cells were fixed in 1% formaldehyde in 1× PHEM with 10 μM microcystin for 12 min at room temperature. Coverslips were then washed with 0.5× PHEM, and immunofluorescence was done as described previously [51]. Images *Z*-stacks were collected using the Scanning Confocal Microscope Leica SP2 AOBS SE (Leica Microsystems) and the software provided by the manufacturer, Software LCS (Leica Microsystems). Data stacks were deconvolved, using the Huygens Essential version 3.0.2p1 (Scientific Volume Imaging).

### Immunofluorescence in HeLa cells.

HeLa cells were cultured in DMEM medium (Invitrogen) supplemented with 10% fetal bovine serum (FBS) and grown at 37 °C in a 5% CO_2_ humidified chamber. Cells were fixed for 12 min in freshly prepared 2% paraformaldehyde (Sigma) in 1× PHEM, permeabilized with 0.5% Triton X-100 in PBS 3 times for 5 min, washed in PBS, and blocked with 10% FBS. Incubation with primary and secondary antibodies was performed in 1× PBS with 10% FBS.

### Antibodies.

Primary antibodies were anti–α-tubulin (mouse mAb B512), used at 1:3,000 (Sigma-Aldrich); antiphosphorylated histone H3 rabbit polyclonal, used at 1:500 (Upstate Biotechnology); anti-CID chicken polyclonal, used at 1:200 [52]; anti-SMC4 antibodies (rabbit, 1:500, or sheep, 1:200), as described previously [53]; anti-Bubr1 rat polyclonal [25] used at 1:3,000; anti-RAD21 [29] rabbit polyclonal (1:500); anti-Polo mouse mAb MA294, used at 1:50 [54]; anti–Aurora B polyclonal antibody [36], used at 1:1,000; anti–Aurora B polyclonal antibody [55], used at 1:500 in western blot; antiphospho(Ser^7^)CENPA (Upstate Biotechnology), used at 1:500; anti-3F3/2 mAb [56], used at 1:1,000; anti-CENP-meta rabbit polyclonal (Byron Williams) used at 1: 1,000; anti–cyclin B rabbit polyclonal [57], used at 1: 3,000; and anti–TOPO II mouse mAb P2G3 [58], used at 1:20.

### Drug treatments.

MG132 (Sigma) was used to inhibit the proteasome activity in S2 *Drosophila* cells according to the conditions previously described [25]. ICRF-187 at 50 μg/ml was used to inhibit TOPO II activity both in S2 *Drosophila* cells and in HeLa cells. Incubations were always performed for 2 h, excepted for in vivo–timed TOPO II inhibition. The TOPO II inhibition was done according to the description in each figure.

### In situ hybridization.

Fluorescent in situ hybridization to mitotic chromosomes, 2 × 10^5^ cells were centrifuged onto slides, after which cells were simultaneously fixed and extracted as described previously. Cells were dehydrated by incubation for 5 min in 70%, 80%, and 100% ethanol at 4 °C. Cells were air dried and denatured in 2× SSC; 70% formamide for 2 min at 70 °C. Cells were dehydrated once again as described before. We labeled the pericentromeric probe dodeca-satellite DNA with biotin-14-dATP using the BionickTM DNA labeling system (Invitrogen). Detection of the biotinylated probe was done with avidin-D conjugated with fluorescein (Vector Lab).

### Aurora B kinase assay.

A total of 30 ml of S2 cells were grown exponentially to 4–5 × 10^6^ cells/ml, incubated on ice for 45 min, and centrifuged at 1,500*g* for 15 min at 4 °C. Cells were resuspended in 1 ml of cold 1× PBS in the presence of protease inhibitors, kept on ice for 45 min, and lysed in 1 ml of lysis buffer (15 mM Tris-HCl [pH 7.4], 0.2 mM spermine, 0.5 mM spermidine, 2 mM K-EDTA, 1 mM EGTA, 150 mM KCl, 15 mM NaCl, 1 mM DTT, 1% [V/V] Triton X-100) with 2× protease inhibitors-EDTA free (Roche) and 1× phosphatase inhibitor cocktail I (Sigma). Cells were lysed with a B-type pestle in a Dounce homogenizer after which they were incubated on ice for 1 h. Samples were precleared with Protein A-Sepharose CL-4B ( Sigma) for 30 min at 4 °C. Antibody anti–Aurora B (5 μl) and 100 μl of 10% Protein A-Sepharose beads were added to the samples and rotated for 1 h at 4 °C. The beads containing the immune complexes were washed three times in 1 ml of lysis buffer. The pellet was resuspended in 20 μl of serine/threonine kinase assay buffer (10 mM Tris-HCL [pH 7.4]; 0.1% Triton X-100; 10 mM MgCl_2_) with 1× phosphatase inhibitor cocktail I (Sigma), 50 μCi (185 KBq) of [γ^32^P]-adenosine 5′-triphosphate (ATP; >5,000 Ci/mmol; Amersham) and 10 μg of histone H3 (Upstate Biotechnology).

### Preparation of genomic DNA from S2 cells.

The preparation of the genomic DNA was done using 10^7^ cells both for control and for TOPO II double-stranded RNAi (dsRNAi) cells. Cells were collected and spun down at 1,000*g* for 3 min. Pellets were resuspended in 300 μl of STE (150 mM NaCl; 30 mM Tris-HCl [pH 8.0]; 2 mM EDTA). We added 3 μl of 10% NP40, 30 μl 10%SDS, and 30 μl of 10 mg/ml proteinase K (Sigma) and incubated at 55 °C for 3 h. We performed phenol/chloroform extraction. Aqueous fraction was recovered and extracted once again with chloroform. DNA was precipitated with 20 μl of 5 M NaCl and 400 μl of 100% ethanol. Genomic DNA was digested with the restriction enzyme HindIII (Biolabs) according to the manufacturer's conditions. Electrophoresis of the genomic DNA was performed in a 0.7% agarose gel, and the gel was prepared for standard alkaline Southern blotting. DNA probes were radioactively labeled with [α-32P]dCTP using a multiprime labeling kit (Amersham).

## Supporting Information

Figure S1 Analysis of Centromere Organization after Depletion of TOPO II. Immunolocalization of CID in S2 Cells Treated with Hypotonic Shock prior to Cell Fixation with two CID dots per chromosome (individual channel on the right). (B) In TOPO II–depleted cells, chromosomes are observed as larger clusters of chromatids with interconnecting chromatin and broad CID staining at the primary constriction (individual channel on the right).
Fluorescent in situ hybridization with dodeca-satellite DNA, a heterochromatic pericentromeric sequence, was used to specifically identify chromosome 3 in (C) control or (D–F) TOPO II–depleted cells.
(C) In control cells, we observed two clearly defined regions of hybridization per chromosome that localize close to the primary constriction.
(D) In TOPO II–depleted cells at 96 h, several chromosomes (60%, *n* = 60) exhibit four dodeca-satellite regions of hybridization, suggesting that they correspond to diplochromosomes.
(E and F) In TOPO II–depleted cells, larger clusters of homologous chromosomes can also be observed.
(G–I) Fluorescent in situ hybridization with dodeca-satellite DNA (green and separated white channel) was also performed in asynchronous cells, both in control (G) and TOPO II–depleted cells (H and I). At the metaphase plate, chromosomes can be observed individually either in control (G) or in TOPO II–depleted cells (H). However, in anaphase, unseparated centromeres are observed only in TOPO II–depleted cells (I). Most of the chromatin segregate as unseparated chromatids, although a few diplochromosomes can also be observed.
(J) Southern blot was performed for genomic DNA from control S2 cells and TOPO II–depleted cells 96 h after the addition of dsRNA. The ribosomal DNA (rDNA), a heterochromatic sequence localizing specifically to the X centromere proximal region, was used as probe as well as *Zw10*, a single gene also from the X chromosome. *Zw10* was used as an internal control to normalize for the number of X chromosomes. Intensity of ZW10 and rDNA bands was determined by measuring the mean pixel intensity. The ratio for the intensities obtained for the gene and rDNA sequence is the same for the genomic DNA extracted from control and TOPO II–depleted cells. This result indicates that replication of rDNA occurs at the same ratio as the euchromatic genes, suggesting that heterochromatin replication is not specifically affected in the absence of TOPO II. Scale bar represents 5 μm.
(1.62 MB PDF)

Figure S2 Microtubule–Kinetochore Interaction in TOPO II–Depleted Cells (A and B) Immunofluorescence for α-tubulin (green), CID (red), and DNA (blue) in (A) control and (B) TOPO II–depleted cells subjected to the MG132-Taxol assay.
(C) Quantification shows that a few chromosomes (≤3%; control, *n* = 35 cells; TOPO II dsRNAi, *n* = 38 cells), either in control or TOPO II–depleted cells have mono-oriented chromosomes, whereas most show syntelic attachment. No differences between control and TOPO II–depleted cells were obtained during the time course of the experiment.
(D) Interestingly, in spindles that have not yet collapsed, we were able to observe chromatin bridges between chromosomes, suggesting the presence of catenated DNA between chromosomes. Scale bar represents 5 μm.
(601 KB PDF)

Figure S3 Quantification of Sister Centromere Distance during Progression through Mitosis in TOPO II–Depleted Cells (A) Images from time-lapse recording of S2 cells stably expressing the centromere marker CID-mCherry (red and individual channel on the right) and GFP-α-tubulin. *Z*-stacks were collected in both control and TOPO II–depleted cells.
(B) Quantification of sister centromere distance does not show any difference between control (*n* = 70) and TOPO II–depleted cells at 96 h (*n* = 60) during prophase.
(C) Quantification of sister centromere distance during prometaphase, metaphase, and anaphase from (D) time-lapse images of S2 expressing CID-GFP and histone RFP-H2B. The graph (C) shows that whereas in control cells, intercentromere distance increases continuously, in TOPO II–depleted cells, intercentromere distance never changes even when compared to cells in prophase (see [B]). Scale bar represents 5 μm.
(911 KB PDF)

Figure S4 Characterization of Mitotic Exit after Depletion of TOPO II and RAD21 Progression through mitosis was determined using cyclin B to clearly determine exit from mitosis and also the earlier stages, such as prometaphase (72 h of treatment). Either (A) control or (B) TOPO II– and DRAD21-depleted cells were immunostained for cyclin B (green), CID (red), and DNA (blue). Chromatin lagging is observed in late anaphase of double-depleted cells. Scale bar represents 5 μm.
(709 KB PDF)

Figure S5 In Vivo Analysis of Mitotic Progression after Depletion of RAD21 in S2 Cells (A and B) Progression through mitosis was determined using cyclin B (red) and α-tubulin and DNA (blue) for either (A) control or (B) RAD21-depleted cells. Scale bar represents 5 μm. (B) RAD21-depleted cells are delayed in mitosis, exhibiting separated sister chromatids.
(C) Mitotic index quantification shows no significant differences between control and TOPO II–depleted cells through the time course of the experiment. Although we quantified a delay in a prometaphase-like stage with separated sister chromatids, the percentage of mitotic cells did not increase during the time of depletion.
(D) Immunolocalization of RAD21 (white, separated left channel), SMC4 (red), TOPO II (green), and DNA (white, separated right channel) on S2 mitotic cells treated with hypotonic shock was performed in control or RAD21-depleted cells. In RAD21-depleted cells, sister chromatids remain side by side although cohesin protein is not detected.
(E) Images from videos of S2 cells progressing through mitosis after depletion of RAD21 (Video S12). RAD21-depleted S2 cells stably expressing the centromere marker CID-GFP and histone RFP-H2B. For RAD21-depleted cells, ten cells at 72 h of depletion were recorded. Chromosomes do not align at the metaphase plate, and we observe a limited chromosome/sister chromatid movement during mitosis. Eventually, cells exit mitosis after a long period. Scale bar represents 5 μm.
(2.23 MB PDF)

Figure S6 Characterization of Mitotic Exit of TOPO II–Depleted Cells (A and B) Immunolocalization of cyclin B and CID in (A) control or (B) TOPO II–depleted cells. In both control and TOPO II–depleted cells, cyclin B localizes to the spindle, centromeres, and poles during prometaphase and metaphase. During anaphase, the overall level of cyclin B falls and is almost undetectable by telophase. Cyclin B staining was used to confirm that in the absence of TOPO II, mitotic cells exhibiting either bridges or lagging chromatin were indeed in anaphase, as they exhibit low levels of cyclin B.
(C) Mitotic progression was determined using cyclin B immunolocalization, both at 72 h and 96 h after the addition of dsRNA. Compared to control cells, an increase in prometaphase TOPO II–depleted cells was detected, which parallels a decrease in the percentage of cells in metaphase. Scale bar represents 5 μm.
(1.07 MB PDF)

Figure S7 Immunolocalization of PH3 and TOPO II in ICRF-187–Treated Cells (A) Control and (B) cells treated for 2 h with 10 μM ICRF-187, after which they were immunostained for Phospho-S10-histone H3 (green) and for TOPO II (blue). TOPO II and PH3 localized to the chromosomes both in control and treated cells, although a reduction in the levels of PH3 was detected.
(C) Phospho-S10-histone H3 levels were quantified both in control (*n* = 25) and in ICRF-187–treated cells (*n* = 20). Images were collected in the same conditions, and the mean pixel intensity was determined. The 45% reduction observed in treated cells is significant by the Student *t* test (*p* < 0.005). Scale bar represents 5 μm.
(803 KB PDF)

Video S1 Time-Lapse Confocal Microscopy of S2 Cells Stably Expressing CID-GFP and RFP-H2B *Z*-stacks were acquired every 30 s (time is shown in seconds). Each Z-stack is 10 μm and composed of ten optical sections. Anaphase onset corresponds to time 0 s. Merge color images of RFP-H2B (red) and CID-GFP (green) channels are shown on the left. The CID-GFP (white) channel is shown alone on the right. The two CID-GFP pairs of each centromere were identified and labeled in every layer composing the *Z*-stacks at the different time points. The mean distance between CID-GFP dots is approximately 1 μm. Tracking of kinetochore pairs was performed using a plug-in for the Image J software. The video of each CID-GFP pair is labeled with the same color.
(533 KB MOV)

Video S2 Time-Lapse Microscopy of S2 Cells Stably Expressing CID-GFP and RFP-H2B and Depleted for TOPO II, 72 h after the Addition of the dsRNA *Z*-stacks are composed of ten optical sections covering 10 μm and were acquired every 20 s. Each Z-stack is 10 μm and composed of ten optical sections. Anaphase onset corresponds to time 0 s. Merge color images of RFP-H2B (red) and CID-GFP (green) channels are shown on the left. The CID-GFP (white) channel is shown alone on the right. The two CID-GFP pairs of each centromere were identified and labeled in every layer composing the *Z*-stacks at the different time points. The mean distance between CID-GFP dots is approximately 1 μm. Tracking of kinetochore pairs was performed using a plug-in for the Image J software. In the video, each CID-GFP pair is labeled with the same color.
(1.48 MB MOV)

Video S3 Time-Lapse Microscopy of S2 Cells Stably Expressing CID-GFP and RFP-H2B and Depleted for TOPO II, 72 h after the Addition of the dsRNA *Z*-stacks are composed of ten optical sections covering 10 μm and were acquired every 15 s. Anaphase onset corresponds to time 0 s. Merge color images for H2B- RFP (red) and CID-GFP (green).
(806 KB MOV)

Video S4 Time-Lapse Microscopy of S2 cells Stably Expressing CID-GFP and RFP-H2B and Depleted for TOPO II, 96 h after the Addition of the dsRNA *Z*-stacks are composed of ten optical sections covering 10 μm and were acquired every 15 s. Anaphase onset corresponds to time 0 s. Merge color images for H2BRFP (red) and CID-GFP (green) is shown on the left. The separate channel for CID-GFP (white) can be seen on the right. Pairs of centromeres were identified and labeled in each optical section of the *Z*-stack as described above. Pairs of the centromere marker CID-GFP are labeled with the same color.
(378 KB MOV)

Video S5 Time-Lapse Microscopy of S2 Cells Stably Expressing GFP-α-Tubulin and CID-mCherry *Z*-stacks were acquired every 40 s. Each *Z*-stack is 10 μm and composed of ten optical sections. Anaphase onset corresponds to time 0 s. Merge color images for CID-mCherry (red) and GFP-α-tubulin (green) are shown on the left. On the right, the separated channel for CID-GFP (white) is shown, and CID-GFP pairs are labeled with the same color.
(608 KB MOV)

Video S6 Time-Lapse Microscopy of S2 Cells Stably Expressing GFP-α-Tubulin and CID-mCherry That Were Depleted for TOPO II, 72 h after the Addition of the dsRNA *Z*-stacks were acquired every 40 s. Each *Z*-stack is 10 μm and is composed of ten optical sections. Anaphase onset corresponds to time 0 s. Merged color images for Cherry-CID (red) and GFP-α-tubulin (green) are shown on the left. In the middle, the separated channel for GFP-α-tubulin (white) is shown with representation of the CID pairs composing each centromere (on the right, separated channel).
(582 KB MOV)

Video S7 Time-Lapse Microscopy of S2 Cells Stably Expressing GFP-α-Tubulin and CID-mCherry *Z*-stacks were acquired every 30 s. Each *Z*-stack is 10 μm and composed of ten optical sections. Anaphase onset corresponds to time 0 s. Merge color images for CID-mCherry (red) and GFP-α-tubulin (green) is shown on the left. Separated channels for CID-GFP (black) and GFP-α-tubulin (black) are shown in the middle and on the right, respectively.
(1.19 MB MOV)

Video S8 Time-Lapse Microscopy of S2 Cells Stably Expressing GFP-α-Tubulin and CID-mCherry That Were Incubated with 50 μg/ml ICRF-187 after the Establishment of a Bipolar Attachment *Z*-stacks were acquired every 30 s. Each *Z*-stack is 10 μm and composed of ten optical sections. Merge color images for CID-mCherry (red) and GFP-α-tubulin (green) are shown on the left. Separated channels for CID-GFP (black) and GFP-α-tubulin (black) are shown in the middle and on the right, respectively. The time of ICRF-187 addition to a final concentration of 50 μg/ml is indicated.
(5.24 MB MOV)

Video S9 Time-Lapse Microscopy of S2 Cells Stably Expressing GFP-α-Tubulin and CID-mCherry That Were Incubated for 1 h with 50 μg/ml ICRF-187 before the Recording *Z*-stacks were acquired every 30 s. Each *Z*-stack is 10 μm and composed of ten optical sections. Anaphase onset corresponds to time 0 sec. Merge color images for CID-mCherry (red) and GFP-α-tubulin (green) are shown on the left. Separated channels for CID-GFP (black) and GFP-α-tubulin (black) are shown in the middle and on the right, respectively. The time of ICRF-187 addition to a final concentration of 50 μg/ml is indicated.
(5.81 MB MOV)

Video S10 Time-Lapse Microscopy of S2 Cells Stably Expressing CID-GFP and RFP-H2B That Were Simultaneously Depleted of TOPO II and RAD21, 96 h after the Addition of the dsRNA *Z*-stacks were acquired every 15 s. Each *Z*-stack is 10 μm and is composed of ten optical sections. Anaphase onset corresponds to time 0 s. Merged color images for H2B- RFP (red) and CID-GFP (green) are shown on the left. The separated channel for CID-GFP (white) can be seen on the right. Pairs of centromere marker CID-GFP were identified and labeled on each layer of the *Z*-stacks, and are shown in Video S6. Pairs of CID-GFP dots are labeled with the same color.
(1.84 MB MOV)

Video S11 Time-Lapse Microscopy of S2 Cells Stably Expressing CID-GFP and RFP-H2B That Were Simultaneously Depleted of TOPO II and RAD21, 96 h after the Addition of the dsRNA *Z*-stacks were acquired every 15 s. Each *Z*-stack is 10 μm and is composed of ten optical sections. Anaphase onset corresponds to time 0 s. Merged color images for H2B- RFP (red) and CID-GFP (green) are shown on the left. The separated channel for CID-GFP (white) can be seen on the right. Pairs of centromere marker CID-GFP were identified and labeled on each layer of the *Z*-stacks, and are shown in Video S6. Pairs of CID-GFP dots are labeled with the same color.
(356 KB MOV)

Video S12 Time-Lapse Microscopy of S2 Cells Stably Expressing CID-GFP and RFP-H2B That Were Depleted of RAD21, 96 h after the Addition of the dsRNA Each *Z*-stack is 10 μm and is composed of ten optical sections. Anaphase onset corresponds to time 0 s. Merged color images for H2B-RFP (red) and CID-GFP (green) are shown on the left. The separated channel for CID-GFP (white) can be seen on the right.
(2.26 MB MOV)

## Abbreviations

CPC: chromosome passenger complex
dsRNA: double-stranded RNA
dsRNAi: double-stranded RNA interference
PH3: phosphohistone H3 (Ser10)
RNAi: RNA interference
SAC: spindle assembly checkpoint
TOPO II: topoisomerase II
