# Supplementary figures and images for: Dual Role of Topoisomerase II in Centromere Resolution and Aurora B Activity
Source: PLoS Biol. 2008 Aug 26;6(8):e207. doi: 10.1371/journal.pbio.0060207 (PMC2525683; doi:10.1371/journal.pbio.0060207)

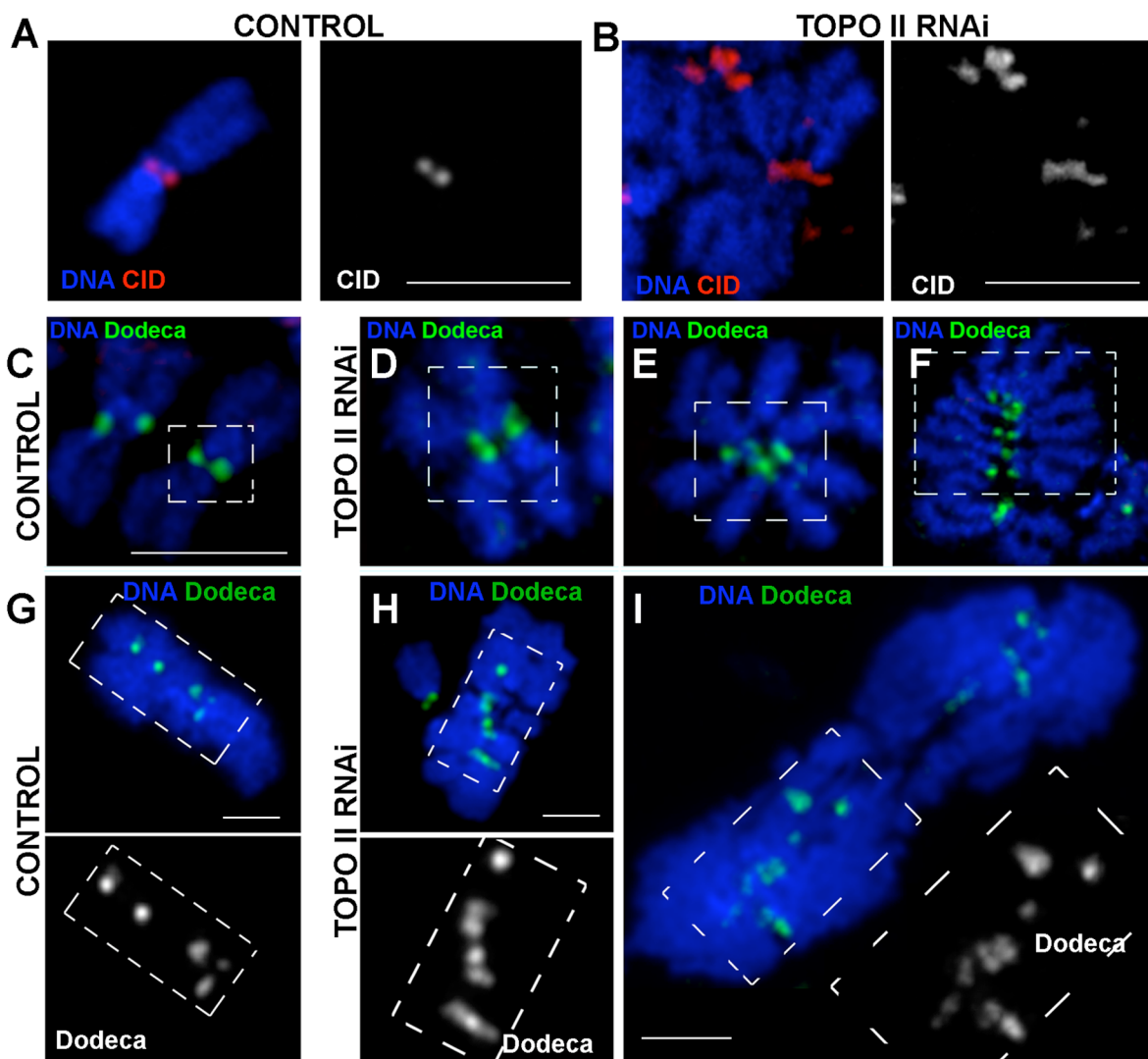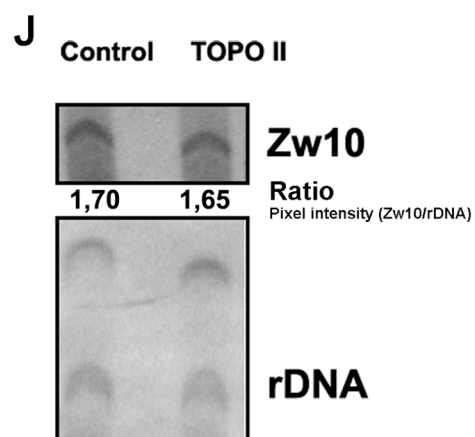

Supplement: Figure S1 — (B) In TOPO II–depleted cells, chromosomes are observed as larger clusters of chromatids with interconnecting chromatin and broad CID staining at the primary constriction (individual channel on the right). Fluorescent in situ hybridization with dodeca-satellite DNA, a heterochromatic pericentromeric sequence, was used to specifically identify chromosome 3 in (C) control or (D–F) TOPO II–depleted cells. (C) In control cells, we observed two clearly defined regions of hybridization per chromosome that localize close to the primary constriction. (D) In TOPO II–depleted cells at 96 h, several chromosomes (60%, n = 60) exhibit four dodeca-satellite regions of hybridization, suggesting that they correspond to diplochromosomes. (E and F) In TOPO II–depleted cells, larger clusters of homologous chromosomes can also be observed. (G–I) Fluorescent in situ hybridization with dodeca-satellite DNA (green and separated white channel) was also performed in asynchronous cells, both in control (G) and TOPO II–depleted cells (H and I). At the metaphase plate, chromosomes can be observed individually either in control (G) or in TOPO II–depleted cells (H). However, in anaphase, unseparated centromeres are observed only in TOPO II–depleted cells (I). Most of the chromatin segregate as unseparated chromatids, although a few diplochromosomes can also be observed. (J) Southern blot was performed for genomic DNA from control S2 cells and TOPO II–depleted cells 96 h after the addition of dsRNA. The ribosomal DNA (rDNA), a heterochromatic sequence localizing specifically to the X centromere proximal region, was used as probe as well as Zw10, a single gene also from the X chromosome. Zw10 was used as an internal control to normalize for the number of X chromosomes. Intensity of ZW10 and rDNA bands was determined by measuring the mean pixel intensity. The ratio for the intensities obtained for the gene and rDNA sequence is the same for the genomic DNA extracted from control and TOPO II–depleted [file pbio.0060207.sg001.pdf]

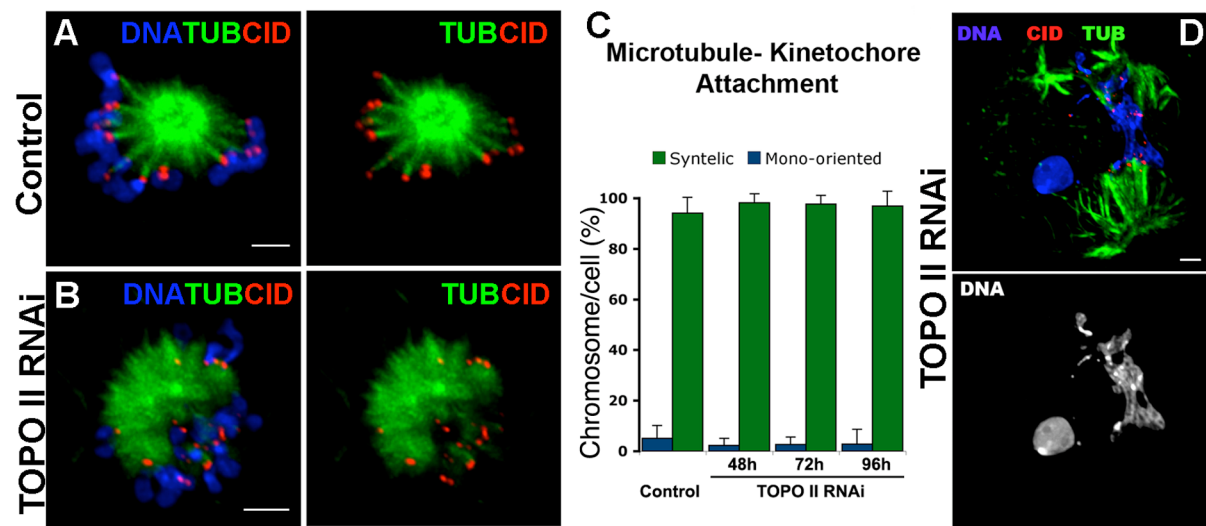

Supplement: Figure S2 — (A and B) Immunofluorescence for α-tubulin (green), CID (red), and DNA (blue) in (A) control and (B) TOPO II–depleted cells subjected to the MG132-Taxol assay. (C) Quantification shows that a few chromosomes (≤3%; control, n = 35 cells; TOPO II dsRNAi, n = 38 cells), either in control or TOPO II–depleted cells have mono-oriented chromosomes, whereas most show syntelic attachment. No differences between control and TOPO II–depleted cells were obtained during the time course of the experiment. (D) Interestingly, in spindles that have not yet collapsed, we were able to observe chromatin bridges between chromosomes, suggesting the presence of catenated DNA between chromosomes. Scale bar represents 5 μm. (601 KB PDF) [file pbio.0060207.sg002.pdf]

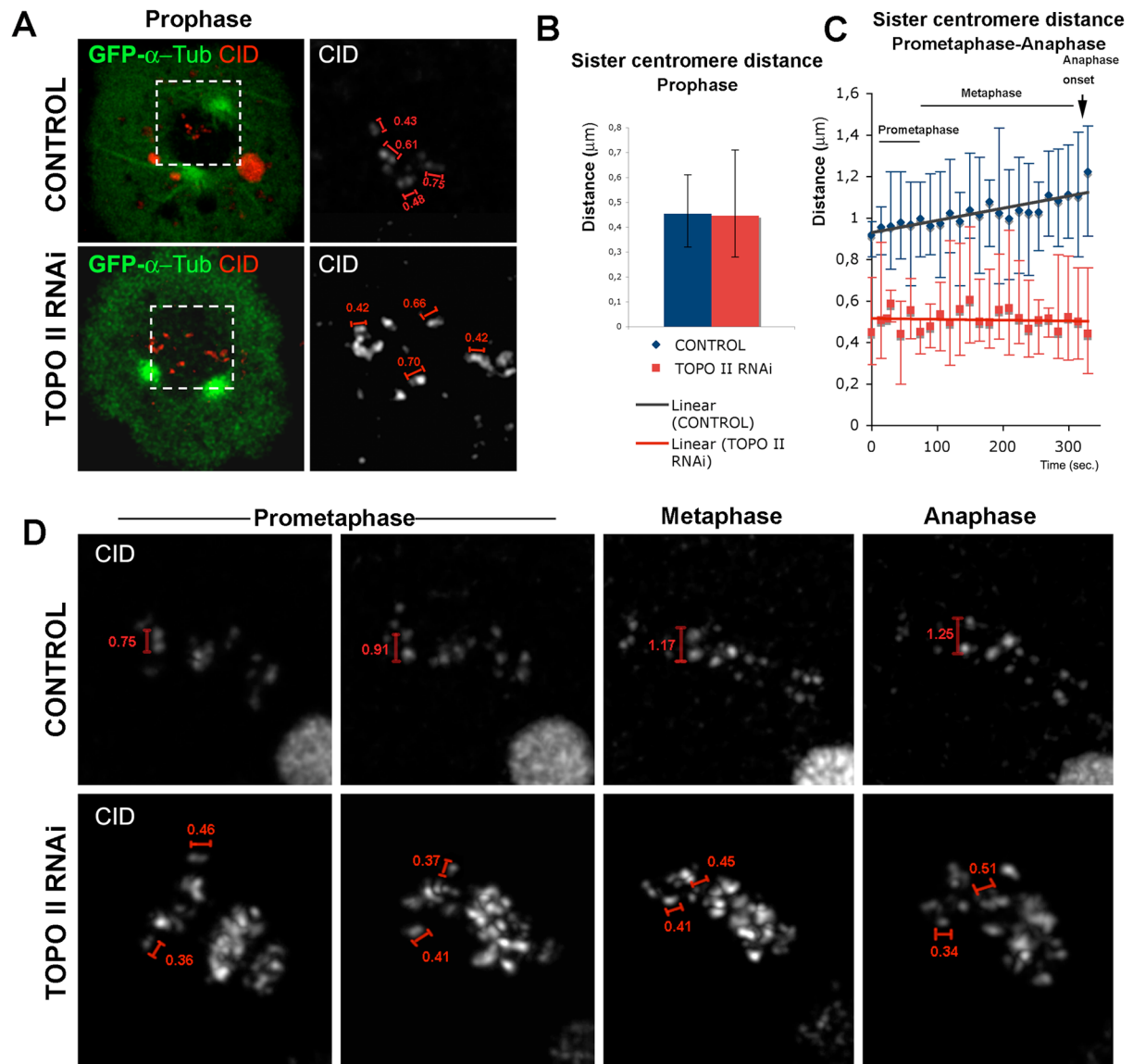

Supplement: Figure S3 — (A) Images from time-lapse recording of S2 cells stably expressing the centromere marker CID-mCherry (red and individual channel on the right) and GFP-α-tubulin. Z-stacks were collected in both control and TOPO II–depleted cells. (B) Quantification of sister centromere distance does not show any difference between control (n = 70) and TOPO II–depleted cells at 96 h (n = 60) during prophase. (C) Quantification of sister centromere distance during prometaphase, metaphase, and anaphase from (D) time-lapse images of S2 expressing CID-GFP and histone RFP-H2B. The graph (C) shows that whereas in control cells, intercentromere distance increases continuously, in TOPO II–depleted cells, intercentromere distance never changes even when compared to cells in prophase (see [B]). Scale bar represents 5 μm. (911 KB PDF) [file pbio.0060207.sg003.pdf]

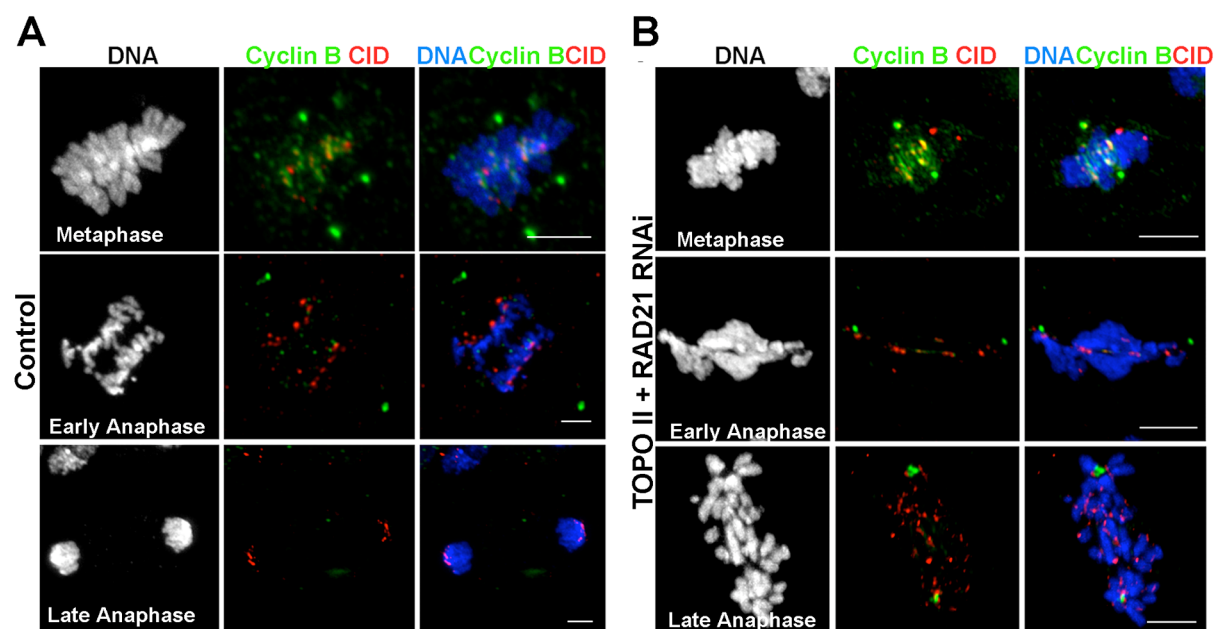

Supplement: Figure S4 — Progression through mitosis was determined using cyclin B to clearly determine exit from mitosis and also the earlier stages, such as prometaphase (72 h of treatment). Either (A) control or (B) TOPO II– and DRAD21-depleted cells were immunostained for cyclin B (green), CID (red), and DNA (blue). Chromatin lagging is observed in late anaphase of double-depleted cells. Scale bar represents 5 μm. (709 KB PDF) [file pbio.0060207.sg004.pdf]

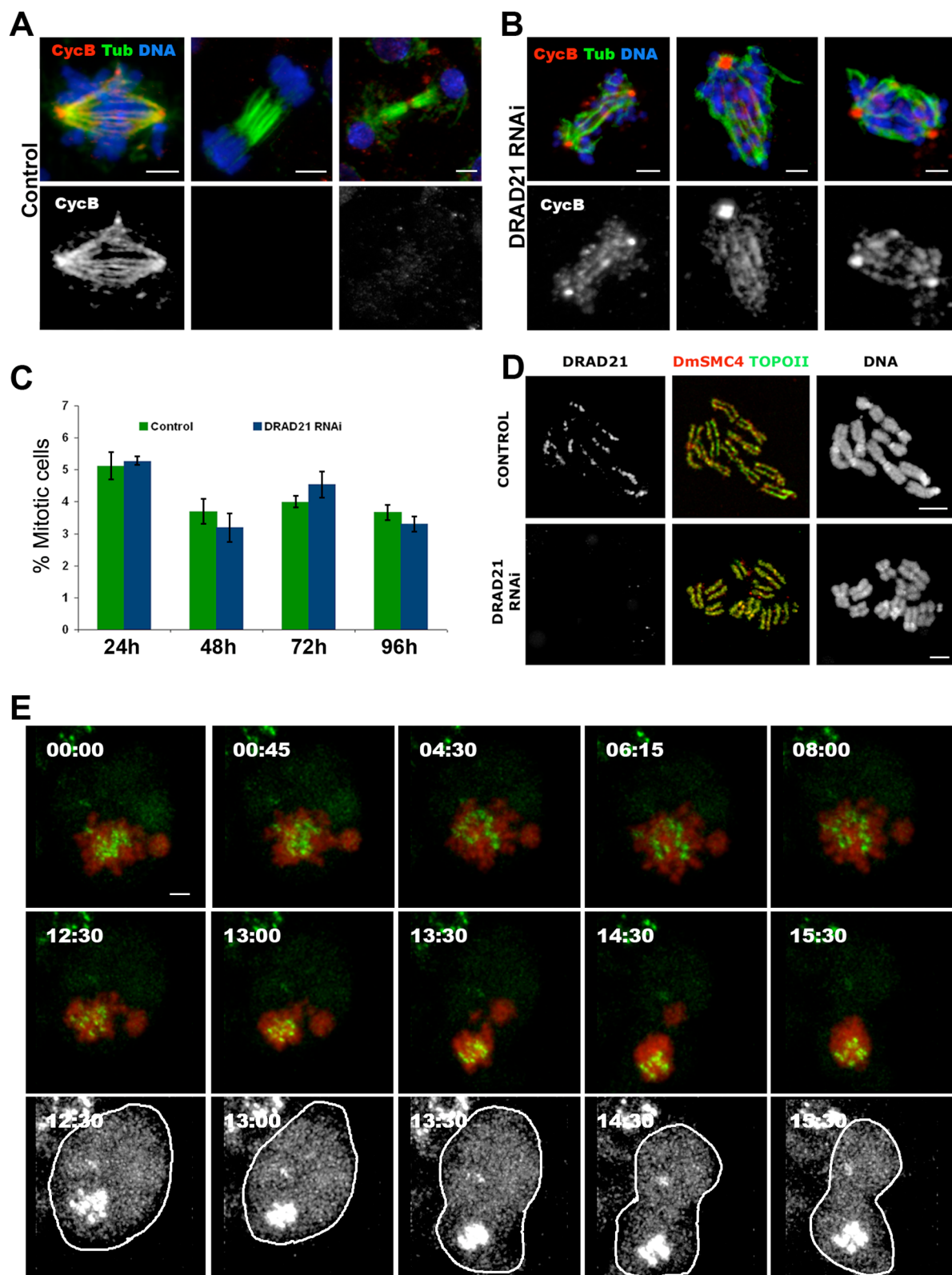

Supplement: Figure S5 — (A and B) Progression through mitosis was determined using cyclin B (red) and α-tubulin and DNA (blue) for either (A) control or (B) RAD21-depleted cells. Scale bar represents 5 μm. (B) RAD21-depleted cells are delayed in mitosis, exhibiting separated sister chromatids. (C) Mitotic index quantification shows no significant differences between control and TOPO II–depleted cells through the time course of the experiment. Although we quantified a delay in a prometaphase-like stage with separated sister chromatids, the percentage of mitotic cells did not increase during the time of depletion. (D) Immunolocalization of RAD21 (white, separated left channel), SMC4 (red), TOPO II (green), and DNA (white, separated right channel) on S2 mitotic cells treated with hypotonic shock was performed in control or RAD21-depleted cells. In RAD21-depleted cells, sister chromatids remain side by side although cohesin protein is not detected. (E) Images from videos of S2 cells progressing through mitosis after depletion of RAD21 (Video S12). RAD21-depleted S2 cells stably expressing the centromere marker CID-GFP and histone RFP-H2B. For RAD21-depleted cells, ten cells at 72 h of depletion were recorded. Chromosomes do not align at the metaphase plate, and we observe a limited chromosome/sister chromatid movement during mitosis. Eventually, cells exit mitosis after a long period. Scale bar represents 5 μm. (2.23 MB PDF) [file pbio.0060207.sg005.pdf]

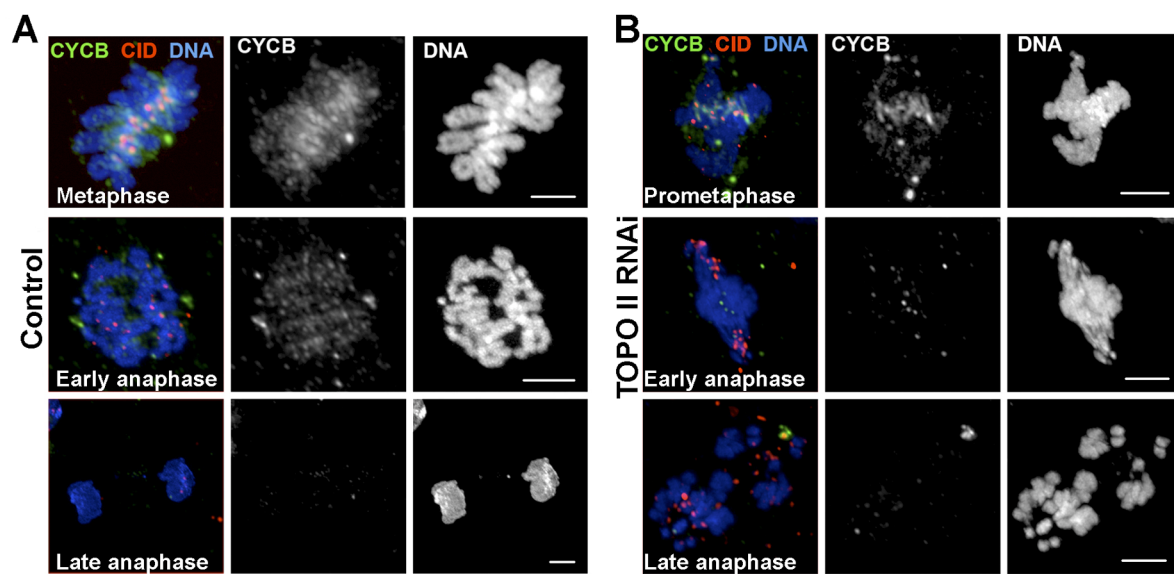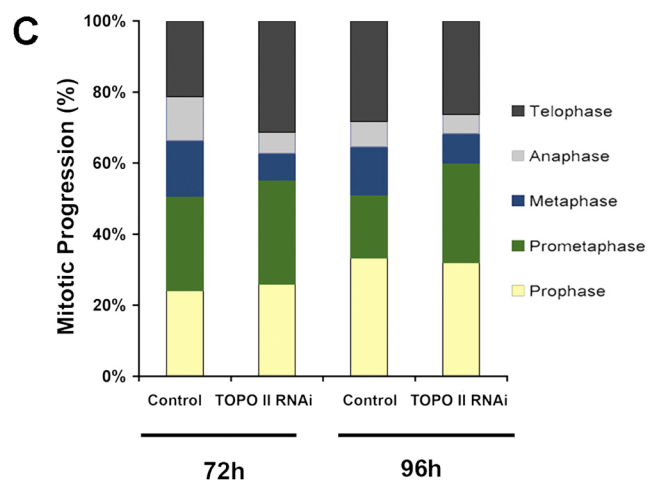

Supplement: Figure S6 — (A and B) Immunolocalization of cyclin B and CID in (A) control or (B) TOPO II–depleted cells. In both control and TOPO II–depleted cells, cyclin B localizes to the spindle, centromeres, and poles during prometaphase and metaphase. During anaphase, the overall level of cyclin B falls and is almost undetectable by telophase. Cyclin B staining was used to confirm that in the absence of TOPO II, mitotic cells exhibiting either bridges or lagging chromatin were indeed in anaphase, as they exhibit low levels of cyclin B. (C) Mitotic progression was determined using cyclin B immunolocalization, both at 72 h and 96 h after the addition of dsRNA. Compared to control cells, an increase in prometaphase TOPO II–depleted cells was detected, which parallels a decrease in the percentage of cells in metaphase. Scale bar represents 5 μm. (1.07 MB PDF) [file pbio.0060207.sg006.pdf]

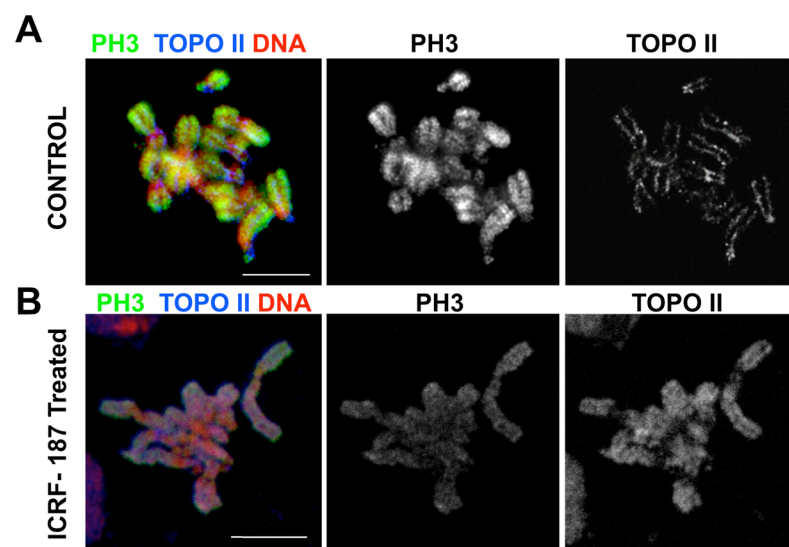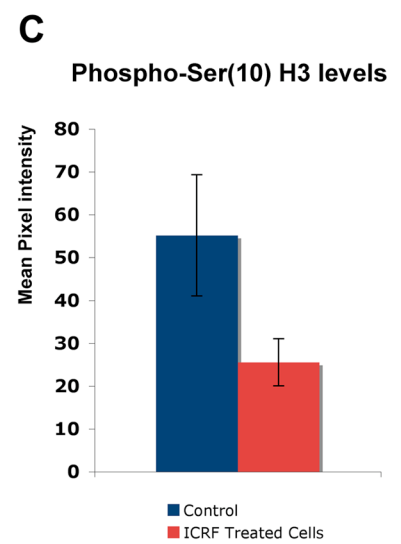

Supplement: Figure S7 — (A) Control and (B) cells treated for 2 h with 10 μM ICRF-187, after which they were immunostained for Phospho-S10-histone H3 (green) and for TOPO II (blue). TOPO II and PH3 localized to the chromosomes both in control and treated cells, although a reduction in the levels of PH3 was detected. (C) Phospho-S10-histone H3 levels were quantified both in control (n = 25) and in ICRF-187–treated cells (n = 20). Images were collected in the same conditions, and the mean pixel intensity was determined. The 45% reduction observed in treated cells is significant by the Student t test (p < 0.005). Scale bar represents 5 μm. (803 KB PDF) [file pbio.0060207.sg007.pdf]
